# Neural activity patterns in the chemosensory network encoding vomeronasal and olfactory information in mice

**DOI:** 10.3389/fnana.2022.988015

**Published:** 2022-09-02

**Authors:** Cecília Pardo-Bellver, Manuel E. Vila-Martin, Sergio Martínez-Bellver, María Villafranca-Faus, Anna Teruel-Sanchis, Camila A. Savarelli-Balsamo, Sylwia M. Drabik, Joana Martínez-Ricós, Ana Cervera-Ferri, Fernando Martínez-García, Enrique Lanuza, Vicent Teruel-Martí

**Affiliations:** ^1^Department of Functional and Cell Biology, Faculty of Biology, University of Valencia, Valencia, Spain; ^2^Laboratory of Neuronal Circuits, Department of Human Anatomy and Embryology, Faculty of Medicine, University of Valencia, Valencia, Spain; ^3^Department of Neurophysiology and Chronobiology, Institute of Zoology, Jagiellonian University, Kraków, Poland; ^4^Faculty of Health Sciences, Pre-Departmental Unit of Medicine, Jaume I University, Castellón de la Plana, Spain

**Keywords:** amygdala, Granger causality, accessory olfactory system, oscillations, c-Fos

## Abstract

Rodents detect chemical information mainly through the olfactory and vomeronasal systems, which play complementary roles to orchestrate appropriate behavioral responses. To characterize the integration of chemosensory information, we have performed electrophysiological and c-Fos studies of the bulbo–amygdalar network in freely behaving female mice exploring neutral or conspecific stimuli. We hypothesize that processing conspecifics stimuli requires both chemosensory systems, and thus our results will show shared patterns of activity in olfactory and vomeronasal structures. Were the hypothesis not true, the activity of the vomeronasal structures would be independent of that of the main olfactory system. In the c-Fos analysis, we assessed the activation elicited by neutral olfactory or male stimuli in a broader network. Male urine induced a significantly higher activity in the vomeronasal system compared to that induced by a neutral odorant. Concerning the olfactory system, only the cortex–amygdala transition area showed significant activation. No differential c-Fos expression was found in the reward system and the basolateral amygdala. These functional patterns in the chemosensory circuitry reveal a strong top-down control of the amygdala over both olfactory bulbs, suggesting an active role of the amygdala in the integration of chemosensory information directing the activity of the bulbs during environmental exploration.

## Introduction

In the 1970s, the anatomical description of the projections from the vomeronasal organ to the accessory olfactory bulbs and, in turn, to the amygdala (Winans and Scalia, 1970), suggested the idea of an accessory olfactory system processing information in parallel to the main olfactory system (Skeen and Hall, 1977). Although a small degree of anatomical convergence of the projections of both bulbs in the medial amygdala was described in the same years (Scalia and Winans, 1975), most of the target areas are different. The main olfactory bulb (MOB) projects mainly to the anterior olfactory nucleus, the olfactory tubercle, the piriform and entorhinal cortices, and some amygdaloid structures (nucleus of the lateral olfactory tract, anterior, and posterolateral cortical amygdala). In contrast, the accessory olfactory bulb (AOB) projects exclusively to the medial and posteromedial cortical amygdala and part of the bed nucleus of the stria terminalis. As above-mentioned, the medial amygdala and some other amygdaloid nuclei receive sparse common inputs from both systems (Gutiérrez-Castellanos et al., 2010). However, from the anatomical point of view, the level of convergence is minimal.

In sharp contrast with this anatomical view of separated olfactory and vomeronasal systems, functional data always pointed to a complementary role of both chemosensory systems. The first study of the effects of lesions of the olfactory and vomeronasal systems on sexual behavior in hamsters showed that lesioning both systems resulted in much more severe deficits than lesioning only one of them (Powers and Winans, 1975). The exact contribution of each chemosensory system is still a matter of debate, but many functional studies have confirmed its complementary function (Baum and Kelliher, 2009; Keller et al., 2009; Martínez-García et al., 2009). To elucidate the contribution of both systems in processing chemical signals, we analyze the activity in the bulbo–amygdalar network in female mice exploring either neutral olfactory stimuli or conspecifics stimuli, including olfactory and vomeronasal cues. Our hypothesis is that processing conspecifics stimuli requires both chemosensory systems, and thus will induce common patterns of activity in olfactory and vomeronasal structures. If this hypothesis is not true, the activity of the vomeronasal structures would be independent from that of the main olfactory system.

To test this hypothesis, we used a dual approach: the study of the chemosensory network with electrophysiological methods and the anatomical study of neural activity revealed by c-Fos expression to better understand the entire circuitry involved in this process. Neural encoding has been associated with oscillations of the local field potential (LFP), which reflect the weighted average of synchronized activity of neuronal assemblies associated with information processing. This concurrent activity would favor temporal coordination of the incoming sensory information facilitating its transfer across regions (Varela et al., 2001; Siegel et al., 2012). We characterized the oscillatory profiles and their relationship with concurrent higher frequencies by recording electrodes placed in MOB, AOB, medial amygdala (Me), and posteromedial cortical amygdala (PMCo). In the electrophysiological experiments, LFPs in awake freely moving animals were recorded while exploring olfactory stimuli (clean bedding or geraniol) or mixed olfactory-vomeronasal stimuli derived from conspecifics (bedding soiled by females, castrated males or intact males). The flow of information in the bulbo–amygdalar network was analyzed using the Granger causality. These recording sites allow us to characterize the pattern of oscillatory activity in the MOB and AOB, which are the input structures to the main and accessory olfactory systems, respectively, in the Me, which is the principal node receiving direct projections from the MOB and AOB, and finally in PMCo, which is considered the vomeronasal cortex. Furthermore, we detected the increase in the complexity of the oscillatory patterns within these areas by studying the theta nested spectral components, and relating these components with the causality spectral signatures.

The LFP analysis allows us to characterize the temporal dynamics of the activity induced by the different stimuli but in a limited number of structures. Thus, we complemented the study with an anatomical analysis of c-Fos expression as a marker of neural activity in an extended version of the same circuit, including the piriform cortex, other nuclei of the chemosensory amygdala, and the brain reward system.

## Materials and methods

### Subjects

To study the electrical activity in the bulbo–amygdalar network, we used nine adults, virgin female mice (*Mus musculus*) of the CD1 strain (Janvier, France). Adult mice of the same strain provided soiled bedding material (soft wood shavings, Souralit S.L., ref. 3000, Spain). To assess the c-Fos expression in the chemosensory systems and extended reward circuit under an olfactory-vomeronasal behavioral paradigm, we used 10 adult CD1 female mice obtained from an official supplier (Animal Facility of the Central Unit of Research, University of Valencia, Spain). For both experiments, we used virgin female mice aged 4–7 months, weighing between 30 and 35 g, in which the estrous cycle was not controlled. Since our study was focused on sociosexual chemosensory communication, we only used females as it is well-established that male-soiled bedding contains male sexual pheromones that are attractive to them (Martínez-Ricós et al., 2007; Roberts et al., 2010).

All animals were housed in individual cages (22 cm × 22 cm × 15 cm) 1 day prior to the beginning of the habituation and kept on a 12-h light/dark cycle with constant room temperature (22 ± 1°C) and humidity during the rest of the experiment, with *ad libitum* access to food and water.

All experimental procedures were approved by the Research Ethics and Animal Welfare Committee of the University of Valencia (A1431417790135, A1283764105250, and A20201118201142) and were under the European Communities Council Directive (2010/63/EU) on the protection of animals used for scientific purposes.

Part of the data from these experiments was published by Pardo-Bellver et al. (2017) and Villafranca-Faus et al. (2021). The surgical procedures, *in vivo* electrophysiological recordings, histological verification of the recording sites, preference test and behavioral analysis, and histological processing are thoroughly described in the aforementioned publications. They are briefly described in the following sections to facilitate manuscript understanding.

### Surgical procedures

**FIGURE 1:**
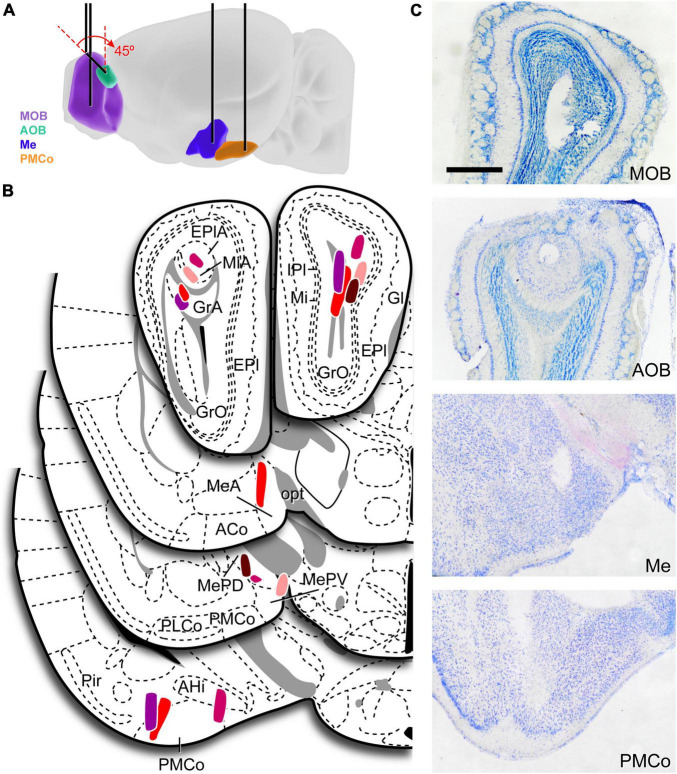
Electrodes implantation and verification. **(A)** Schematic diagram of the recording sites. Black lines represent the monopolar LFP electrodes. **(B)** Schematic drawings of the recording region on all valid cases, each color indicates a different animal (*n* = 5). The number of valid electrodes in each structure is: MOB, *n* = 5; AOB, *n* = 4; Me, *n* = 4; PMCo, *n* = 4. One electrode in the PMCo was located in its caudal part (not shown). **(C)** Histological verification of the electrodes position, the actual recording site (electrode tip) should be along the observed trace. ACo, anterior cortical amygdaloid nucleus; AHi, amygdalo-hippocampal area; AOB, accessory olfactory bulb; EPl, external plexiform layer of the MOB; Gl, glomerular layer of the MOB; GlA, glomerular layer of the AOB; GrA, granular layer of the AOB; GrO, granular layer of the MOB; IPl, internal plexiform layer of the MOB; Me, medial amygaloid nucleus; MeA, anterior medial amygdaloid nucleus; MePD, posterodorsal medial amygdaloid nucleus; MePV, posteroventral medial amygdaloid nucleus; Mi, mitral layer of the MOB; MiA, mitral layer of the AOB; MOB, main olfactory bulb; opt, optic tract; PLCo, posterolateral cortical amygdaloid nucleus; PMCo, posteromedial cortical amygdaloid nucleus.

The electrode implantation was performed under ketamine (75 mg/kg)–medetomidine (1 mg/kg) anesthesia. To record the local field potential, one stainless steel polyimide-coated macroelectrode (E363/6/SPC 0.125 mm, PlasticsOne, United States) was implanted in the AOB (AP: −4.75, L: −1, D: 2.4 mm; at an angle of 45° to the vertical), MOB (AP: −4.75, L: +1, D: 1.3 mm), Me (AP: +1.3 to +1.4, L: −2.1, D: 5.2 mm), and PMCo (AP: +2.7, L: −3, D: 5.6 mm). The stereotaxic coordinates were adapted from Paxinos and Franklin (2004) to CD-1 mice (Figure 1A), the anteroposterior coordinates were measured from Bregma and depth was measured from the cranial surface.

### *In vivo* electrophysiological recordings

The recordings analyzed in this paper have originally been taken from the study by Pardo-Bellver et al. (2017).

All animals were habituated 4 days prior to the recording session for 5 min in an empty methacrylate black opaque box (42.5 cm × 26.5 cm × 18 cm), then they were transferred for 5 min to another methacrylate black opaque box containing clean bedding in a glass plate (6 cm diameter). During the recording session, each animal was successively exposed to the following odor stimuli, always in the same order: two neutral stimuli (clean and geraniol-scented bedding) and three conspecific-derived stimuli (castrated male-, female-, and intact male-soiled bedding). A second recording session was performed a week after the first session. Animals moved freely in the methacrylate boxes. The behavior of the mouse was digitally recorded for offline analysis.

Before the presentation of each stimulus, the mouse was placed in the empty box and a basal period of 20 min was recorded. For the presentation of the stimulus, the mouse was transferred for 5 min to the test box containing a glass plate (6 cm diameter) with 15 ml of the stimulus, to which the animals had full access. After the stimuli presentation, the animal was transferred back to the empty box. The succession of basal and stimulus-presentation periods was repeated until all five stimuli were presented. Two recording sessions were run on different experimental days. In the first session, geraniol and male-derived non-volatile stimuli were novel to the females, whereas clean bedding and female-soiled bedding were familiar to them (from housing at the animal facility). To homogenize these differences among stimuli, between the first and second session, we presented (to all animals) male bedding with geraniol for 4 days. Thus, in the second session, the novelty factor was eliminated. The stimuli were presented in the same order in the second recording session.

Raw signals were amplified and online-filtered between 0.3 and 300 Hz and the 50 Hz noise was analogically removed (p55, Grass Technologies; Ampli 4G21, CIBERTEC, Spain). Then the signals were digitized (Power 1401; Cambridge Electronic Design, United Kingdom) for offline analysis (400 Hz sampling frequency). The waveforms were recorded and continually monitored online using Spike 2 software (Cambridge Electronics Design).

### Histological verification of the recording sites

After the last recording session, the animals were deeply anesthetized with sodium pentobarbital (100 mg/kg; Dolethal Vetoquinol, Spain) and transcardially perfused with saline solution (0.9%) followed by 4% paraformaldehyde (PFA) diluted in phosphate buffer (PB, 0.1 M, pH 7.6). Then, brains were removed, postfixed overnight in the same fixative and cryoprotected in 30% sucrose in PB (0.1 M, pH 7.6) at 4°C until they sank. We used a freezing microtome to obtain coronal sections (40 μm), which were collected in four parallel series. Sections were counterstained with Nissl staining to verify the placement of the electrodes. Attending to our aim, only those cases in which the electrode tip was clearly positioned within the boundaries in at least three of the target areas were included in the study (*n* = 5; Figures 1B,C).

### Electrophysiological experiment: Data analysis

#### Behavioral analysis

Behavioral recordings (see Pardo-Bellver et al., 2017) were reanalyzed with DeepLabCut (version 2.2.0.6), an open-source software for animal pose estimation (Mathis et al., 2018). Twenty frames were extracted from 12 randomly selected videos using k-means clustering to represent the behavioral diversity observed. A network was trained to detect specific mouse body points (see Villafranca-Faus et al., 2021) and the center and periphery of the glass plate in the selected frames for 600000 iterations within the computer cluster of the Bioinformatics and Biostatistics Unit in Principe Felipe Research Centre (CIPF, Spain), achieving a train error of 2.07 pixels and a test error of 12.24 pixels. In the case of error tracking, the network was refined using the same number of iterations, achieving a new train error of 2.42 pixels, and a test error of 5.0 pixels.

Subsequent data from DeepLabCut was analyzed with self-written python scripts. A region of interest was defined from the average X-Y coordinates, with a likelihood value >0.9, from the center and the periphery of the glass dish adding 20 pixels to the result to make the assigned region slightly bigger than the glass dish. Exploration was considered positive when the snout was inside the region of interest. We evaluated exploratory behavior for every minute (1–5) to assess habituation through sessions and behavioral tasks.

To ensure that the mice were actively exploring the provided stimuli, corresponding to the behaviorally relevant epochs for our study, the videos were visually checked. Only those periods where the animal was in the region of interest and moving the nostrils were considered for the electrophysiological analysis.

#### Causality analysis

Synchronization between neural populations plays a crucial role in information processing in the brain. Measures of coupling between the activity of different neuronal assemblies are a defining feature of functional networks involved in neural processing. Granger’s causality (GC; Granger, 1969) is the most widely used tool for identifying causal relationships between time series. In short, a variable *x*_a_ is said to G-cause a variable *x*_b_ if the past of *x*_a_ includes information that helps predict the future of *x*_b_ (Barnett and Seth, 2014). Thus, GC can be considered a measure of directed functional connectivity between two neural activities, represented by a time series of the voltage measurement.

Briefly, in a multivariate model with distinct nodes, each time series *x*_i_(*t*) can be described individually by an autoregressive model based on lagged observations. For a signal *x*_1_(*t*):


$$x_{1}\left(t\right)=\sum_{i=1}^{\text{p}}a_{\text{i}}x_{1}\left(t-i\right)+E_{1}$$

where *p* is the number of lags (model order, determined according to the statistical Akaike Information Criterion), *a*_i_ represents the linear regression coefficients, and *E*_1_ is the prediction error of the model. Moreover, a predictor of *x*_1_(*t*) can also be defined as a part of the multivariate model, assessing the interaction with another time series (x_2_) of the entire network:


$$x_{1}\left(t\right)=\sum_{i=1}^{\text{p}}a_{\text{i}}\cdot x_{1}\left(t-i\right)+\sum_{i=1}^{\text{p}}b_{\text{i}}\cdot x_{2}\left(t-i\right)+E_{12}$$

Here, if the variance (< >) of the prediction error, *E*_12_, is less than the variance of *E*_1_, then it is an indication of a causal interaction from *x*_1_ (*t*) to *x*_2_ (*t*):


$$G_{1\to 2}=\text{ln}\frac{<E_{12}^{2}>}{<E_{1}^{2}>}$$

The asymmetry in the comparison *G*_1→2_ and *G*_2→1_ indicates the directionality of the causal interaction between both time series.

Here, we use the MATLAB toolbox MVGC-Multivariate Granger Causality developed by Barnett and Seth (2014). In our recordings, we calculated the bidirectional causal values in the time domain over periods of exploration in response to the selected stimuli set.

Likewise, we also determined the spectral causality (frequency domain) in the range of 0–120 Hz to detect the distinct oscillatory profiles defining the interaction between neuronal populations. In the spectral formulation, the total time-domain causality is equal to the sum of spectral components over all frequencies. Statistically, for two simultaneously time series, one series can be called *causal* to the other if we can better predict the second series by incorporating past knowledge of the first one (Wiener, 1956). We utilized a random permutation approach to estimate the significance of causal peaks (Brovelli et al., 2005; Dhamala et al., 2018). Specifically, 200 surrogate signals were generated from the original data by randomizing the phases and thus disrupting the structure of the original signals. The comparison of the original signal with each surrogate signal indicated the significance of the spectral profile.

#### Detection of causal profiles

From the above analysis, we obtained a matrix with the significant causality values for each pair of recording areas in both directions. The matrix contained null values in those causal comparisons in which a recording electrode was lost (to visualize the properly placed electrodes, see Figure 1B). In this manner, we calculated the average of the G-values obtaining the interaction profiles for different stimuli from the possible pairings of areas in the neural network. For each of the two recording sessions, we applied clustering techniques intending to identify similarities between the patterns defined by the causal interactions.

To compare the GC profiles for each stimulus, we used an unsupervised hierarchical clustering based on the Euclidean distances capable of correlating similar patterns. We arranged the matrix containing causal interactions as columns (*n* = 12) in a clustergram. The clustered patterns show near positions attending to their Euclidean distance. A bootstrapping technique was used to assess the accuracy of the results, with probabilities calculated after 1000 iterations. The cophenetic correlation coefficient was used as a measure of how faithfully the clustering preserves the pairwise distances between the original data points.

#### Analysis of theta-nested spectral components

Theta activity in the olfactory systems seems crucial in environment exploration (Wachowiak, 2011). The previous analysis of this dataset revealed that theta-gamma patterns in the olfactory systems and amygdala are linked to the integration of the chemosensory information during exploration (Pardo-Bellver et al., 2017). Consequently, we investigate whether the exploration of the stimuli was able to trigger an increase in the theta-coupled processing of the chemosensory network.

We analyzed the presence of theta-nested spectral components (tSCs; Lopes-Dos-Santos et al., 2018) in the MOB, AOB, Me, and PMCo. Briefly, we first applied the empirical mode decomposition, where the signal was decomposed into a set of intrinsic mode functions. For the detection of the main tSCs, we combined the signals in two experimental groups: clean and geraniol-scented bedding (neutral) and female-, castrated and intact male-soiled bedding (conspecific). For each brain region and experimental group, we extracted the theta-band oscillations by merging the intrinsic mode functions derived from the Hilbert–Huang transform, with mean instantaneous frequencies between 3 and 10 Hz. Theta cycles were identified by local maxima and minima of the theta time series. A theta cycle was defined as a sinusoidal wave with a period between 100 ms (10 Hz) and 333 ms (3 Hz), where the zero-phase corresponds to the first trough of the wave. Next, the wavelet power spectrum of the supra-theta signal [10–120 Hz] for each theta cycle was calculated. The spectral signature of each theta cycle was defined as the mean amplitude of the spectrogram in the frequency domain. By principal component analysis, we extracted the first five principal components of the complete set of spectral signatures, representing over 90% of the variance. The procedure continued with extracting the independent components (FastICA algorithm) of the previous principal components, thus recovering the original structure of the spectral signatures. The independent components were defined as tSCs (tSC1 to tSC5) and are the canonical representatives of the set of spectral signatures. To classify each theta cycle in a tSC, we defined the strength of each tSC as the similarity (inner product) between its spectral signature and the representative tSC.

#### Causal – Theta component comparisons

To relate nested theta activity to causal patterns, we performed an additional analysis comparing tSCs with causality spectra for both sets of stimuli. The similarity between two spectral profiles can be represented by a value of vectorial distance. To this end, we computed the *cosine similarity*, a measure using the cosine of the angle between two vectors, limited to the range of [−1, 1] and interpretable as a correlation coefficient.


$$\cos\left(x,y\right)=\frac{x\cdot y}{\left|x\right|\cdot \left|y\right|}$$

Where *x* and *y* are the causal spectral signature and the tSC, respectively. Thus, the larger the similarity value, the closer the two spectral profiles are. In our estimation, each causal direction between structures was compared with the tSCs of the area acting as the source of causality. We selected the 0.9 quantile of the distribution of similarities as a threshold of significance.

### Preference test and behavioral analysis

The c-Fos experiment was performed on freely behaving CD1 female mice exposed to adult intact male urine (*n* = 5) or citralva (*n* = 5), as a neutral odorant (see Villafranca-Faus et al., 2021). To reduce novelty on test day, animals were exposed to volatiles of citralva or male urine during three consecutive days of 10 min habituation sessions. The preference test consisted of a 90 min exposure to 5 g of clean bedding impregnated either with 30 μl of adult male urine (CD1 male mice housed in a different room) or 0.5 μl of citralva, presented in open glass jars. All behavioral experiments were performed within a sound-attenuated room at stable temperature (23°C) and light conditions (≈ 60 lux).

### Histological processing and cell counting

Ninety minutes after the preference test, animals were transcardially perfused with 40 mL of heparinized saline followed by 80 mL of 4% PFA. Brains were postfixed overnight in the same fixative and cryoprotected in 30% sucrose in 0.1 M PB until they sank. Forty-μm-thick coronal slices were obtained in a freezing microtome and collected in five parallel series. Selected slices were treated with 1% sodium borohydride in Tris-buffered saline (TBS, 0.05 M, pH 7.6), and then blocked with 4% normal goat serum (NGS, G9023, SigmaAldrich) and 0.3% Triton X-100 (Tx-100, PanReac AppliChem) in TBS. Afterward, sections were incubated in blocking solution (2% NGS and 0.3% Tx-100 in TBS) with rabbit anti-c-Fos (SC-52, Santa Cruz BioTech, 1:500) overnight at 4°C. After washing, slices were incubated in blocking solution with Alexa Fluor 488 goat anti-rabbit (111-545-003, Jackson ImmunoResearch, 1:200). Finally, sections were counterstained with 4′,6-diamidino-2-phenylindole (DAPI, 1 μg/ml, Molecular BioProducts) in distilled water for 5 min.

Stained sections were mounted onto gelatinized glass slides and covered with FluorSave™ (Merck Millipore). Two-channel (405 and 488 nm) image files were acquired in a FV1000 confocal microscope (Olympus), maintaining the same excitation and acquisition parameters for all samples and structures. Z-series were obtained using sequential scanning mode. For each stack, 6–10 optical sections of 1.94 μm were captured.

Quantitative data were obtained by automated image analysis with a self-written FIJI macro (V2.1.0/1.53c; Schindelin et al., 2012). Macro ran the following steps: (i) enhance contrast in c-Fos channel (488 nm) by increasing the brightness difference between objects and background by 0.2%; (ii) a 0.1% threshold of the highest intensity value for each image was applied, obtaining a 16-bit image; (iii) median filter was applied with *Despeckle* tool; (iv) pixels were added to the edges of objects with *Dilate* tool; (v) noisy pixels were removed with the tool *Remove outliers*; (vi) thresholded particles above 10 pixels were measured and numbered with the *3D Objects Counter* tool (v2.0). Counting ROI was chosen using DAPI channel (405 nm) as reference and delineated as a precise polygon according to Paxinos and Franklin’s mouse brain atlas (2004). A blind experimenter performed nuclei delimitation. Detected objects and ROI areas were automatically saved and results were expressed as cell density of positively labeled cells by normalized area. The cell counting method differs from that used by Villafranca-Faus et al. (2021), so the quantification performed in PMCo differs from the previously reported in terms of absolute density.

### Principal component analysis

Principal component analysis (PCA) was held as an exploratory data analysis to facilitate the interpretation of global c-Fos results. This approach narrows down the original set of variables (immunoreactive-cell density for each nucleus) into a few dimensions or principal components. These components condense most of the variance from the original data, thus reducing overall noise in the model. Since our dataset shows large variance differences among variables, PCA was performed after z-score normalization. Two principal components were selected, as they descriptively cluster our experimental groups while explaining 61.0% of the total variance (PC1 46.5% and PC2 14.5%). To increase the robustness of the PCA, fast-minimum covariance determinant, orthogonalized Gnanadesikan-Kettenring, and Olive-Hawkins estimates were implemented. All of them are based on the Mahalanobis distance for robust outlier detection on multivariate data. Outliers were left in the analysis.

### Statistical analysis

Statistical analysis was performed in RStudio (v4.0.5) and MATLAB (The MathWorks Inc., v2022b).

For behavioral analysis, comparisons of the exploration time between sessions and stimuli were assessed with linear mixed models for repeated measures data. Same statistical model was used to evaluate habituation between first and last minute in each session. Tukey’s range test was performed for multiple comparison.

For the GC and tSC analysis comparisons between non-exploration and active exploration values, we used *t*-test or Wilcoxon’s test, depending on whether the data fulfilled normality (Shapiro-Wilks normality test; *p* < 0.05 to reject) and homoscedasticity (Levene’s test; *p* < 0.05 to reject) conditions.

For the c-Fos experiments, individual comparisons between groups were made using parametric *t*-test or non-parametric Mann-Whitney *U* test, depending on whether the data fulfilled normality and homoscedasticity (Shapiro-Wilks and Levene’s tests) conditions. To measure statistical correlations between variables, we used Pearson’s correlation test. Principal component analysis was performed after z-score normalization by the covariance method. The minimum significance level for all the tests was 0.05.

## Results

**FIGURE 2:**
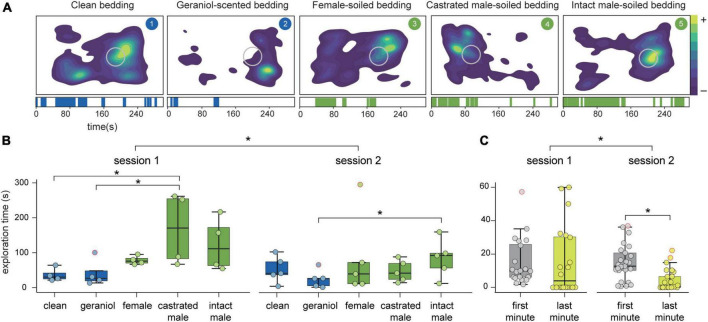
Behavioral analysis. **(A)** Top: density plots of exploration of each stimulus from a representative animal. Region of interest is marked as a gray circle. Down: corresponding ethograms for each stimuli showing positive exploratory events through time (s). **(B)** Average exploration time (s) of each stimulus in each session and **(C)** in the first and last minute of each session. *p < 0.05. Box plots are defined in terms of minima and maxima by whiskers, and the center and bounds of box by quartiles (Q1–Q3). Outliers (dots with red border) were identified by Tukey method and left in the analysis.

We first reanalyzed the behavior of the animals with a deep-learning-based pose-estimation method (Figure 2A). This analysis showed that exploratory behavior differed both between stimuli (*F*_4_ = 5.28, *p* = 0.002) and sessions (*F*_1_ = 9.08, *p* = 0.005; Figure 2B). Multiple comparisons revealed that exploration time of both clean and geraniol-scented bedding differed from castrated and intact male-soiled bedding (clean-castrated male: *p* = 0.04; clean-intact male: *p* = 0.058; geraniol-castrated male: *p* = 0.011; geraniol-intact male: *p* = 0.017), but none of these stimuli showed significant differences with the exploration time of female-scented bedding. By analyzing each session separately, we observed significant differences between stimuli in session 1 (*F*_4_ = 3.84, *p* = 0.031) and a trend in session 2 (*F*_4_ = 2.70, *p* = 0.07). Multiple comparison in session 1 reveals differences between castrated male soiled-bedding and both neutral stimuli (clean-castrated male: *p* = 0.01; geraniol-castrated male: *p* = 0.015). Whereas during session 2, significant differences are only shown between intact male-soiled bedding and geraniol scent bedding (*p* = 0.013).

Differences observed between sessions revealed a decrease in exploratory behavior in session 2, suggesting habituation of animals to the behavioral paradigm. To further assess whether animals were habituated, we compared exploratory time during the first and last minute in each recording session (Figure 2C). Our results showed a significant decrease in exploratory time through sessions (*F*_1_ = 4.38, *p* = 0.039) and a trend to significantly decrease from first to last minute (*F_1_* = 3.54, *p* = 0.063). A trend on the interaction between sessions and time (first-last minute) was also observed (*F*_1_ = 3.72, *p* = 0.057). If we independently compare both sessions with multiple comparison, significant differences between the first and last minute in session 2 (*p* = 0.027), but not in session 1 (*p* = 1.00), were found.

Individual assessment of habituation to each stimuli showed that habituation was significantly observed for the exploration of geraniol (*F*_1_ = 7.56, *p* = 0.015), whereas for the rest of stimuli no significant decrease in exploratory behavior was present between sessions or time (clean: session, *F*_1_ = 0.05, *p* = 0.83 and time, *F*_1_ = 0.11, *p* = 0.75; female: session, *F*_1_ = 1.38, *p* = 0.26 and time *F*_1_ = 0.09, *p* = 0.76; castrated male: session, *F*_1_ = 3.04, *p* = 0.11 and time *F*_1_ = 3.69, *p* = 0.08; intact male: session, *F*_1_ = 0.73, *p* = 0.41 and time *F*_1_ = 1.03, *p* = 0.33).

### The transfer of information between nuclei shows a leading role of the amygdala

Our prior work evidenced synchronic and correlated activity between the theta oscillations in the bulbo–amygdalar network and a phase–amplitude coupling among different frequency bands (Pardo-Bellver et al., 2017). We now attempt to describe the transfer of information within this circuit to clarify the interaction between neuronal groups in the presence of incoming stimuli.

**FIGURE 3:**
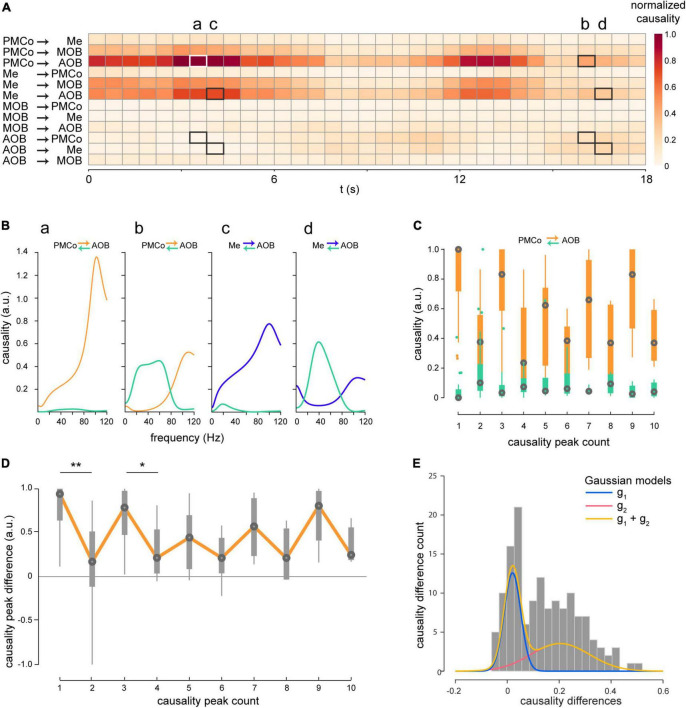
Time-course of the Granger’s causality. **(A)** Heatmap of a representative case of the causal values in the time domain within pairs of brain areas triggered by male-soiled bedding in session 2. (a to d marks) **(B)** Specific segments marked in **(A)** were analyzed in the frequency domain: **(a)** PMCo →AOB, **(b)** AOB →PMCo, **(c)** Me →AOB, **(d)** AOB → Me. **(C)** Boxplot of causality peaks in both directions on consecutive epochs for diverse exploration segments. Note the different positions of the aligned orange and green boxplot as indicative of the difference between causal peaks for both directions. **(D)** Boxplot of the differences between causal peaks derived from **(B,C)**. Boxes represent the difference between consecutive peaks of causality. The orange line is intended to illustrate the cyclic progress of the difference in the causal relationship between PMCo and AOB. The statistical comparisons are made among pairs of consecutive values (*p < 0.05, **p < 0.01). **(E)** Gaussian models of the distribution of the differences in causal values between PMCo and AOB are represented in **(B)**. The orange curve defines the bimodal model, and the blue and red curves the constitutive unimodal models. Akaike’s information criterion confirmed the choice of a dual Gaussian model to represent the data.

The LFP signals of each recording channel were segmented in behaviorally relevant epochs, where the mice were actively exploring the provided stimuli. From the time series, we first quantified Granger’s causal relationship within pairs of restricted brain areas in time and frequency domains (Figure 3). As some epochs presented long exploration times, the analysis windows were divided into shorter overlapping (75%) segments (2 s), thus achieving a detailed view of the directional components between brain areas that are reciprocally interconnected (Pardo-Bellver et al., 2012; Cádiz-Moretti et al., 2016a). In the time domain, causal analysis reflected significant interactions among nuclei, characterized by alternating directionality of the causal flows between them. The example depicted in Figure 3A illustrates two distinctive directionalities permuting when intact male-derived stimuli trigger the bulbo–amygdalar circuitry as a whole. We observed a leading role of the amygdala in its interaction with the bulbs as the characteristic pattern (Me and PMCo to AOB; Figure 3A, 0–9 s). However, we detected a distinctive transition, albeit with a persistent amygdalar dominance, to an increase in the causality values from the bulbs, accompanied by a decrease in causality from the amygdalar areas (AOB to PMCo and Me; Figure 3A, 9–12 s), i.e., closer causality values when comparing both directions (Figure 3C, values of causality peaks for all animals). In detail, a cycling causal relationship was observed when calculating the differences between the causality values for both directions for consecutive periods for all animals (Figure 3D). When we compared consecutive peaks, significant values of differences between both directions (PMCo-AOB) were found (Figure 3D; comparisons between peaks 1 and 2: W = 2.41, *p* = 0.016; comparisons between peaks 3 and 4: W = 3.43, *p* = 0.0006). Due to the low number of peaks, the comparisons beyond the third-four peak did not show significance. For this reason, we modeled the distribution of these differences with Gaussian curves (Figure 3E) in unimodal and bimodal variants. Using the Akaike information criterion, the best fitting model was represented by a bimodal distribution, i.e., two sets of points representing the differences between the causality peaks in consecutive periods (Figure 3E; g_1_: 0.02 ± 0.032 a.u; g_2_: 0.20 ± 0.11 a.u.). Consequently, a cyclical pattern of causal interaction is recognized in the transfer of information in the bulbo–amygdalar circuitry. This phenomenon is not only observed for intact male-soiled bedding, but for all studied stimuli (Supplementary Figure 1).

The causal analysis in the frequency domain showed the dynamics of these connections. During the periods of predominant PMCo→AOB flow, causal peaks were detected in the high-gamma band (Figure 3Ba), whereas in the AOB→PMCo direction, they were present in beta and low gamma frequencies (Figure 3Bb). Similarly, the dominant Me→AOB flow showed a causal peak in the high-gamma band (Figure 3Bc), while the evoked periods revealed causal beta-low gamma peaks in the AOB → Me direction (Figure 3Bd).

**FIGURE 4:**
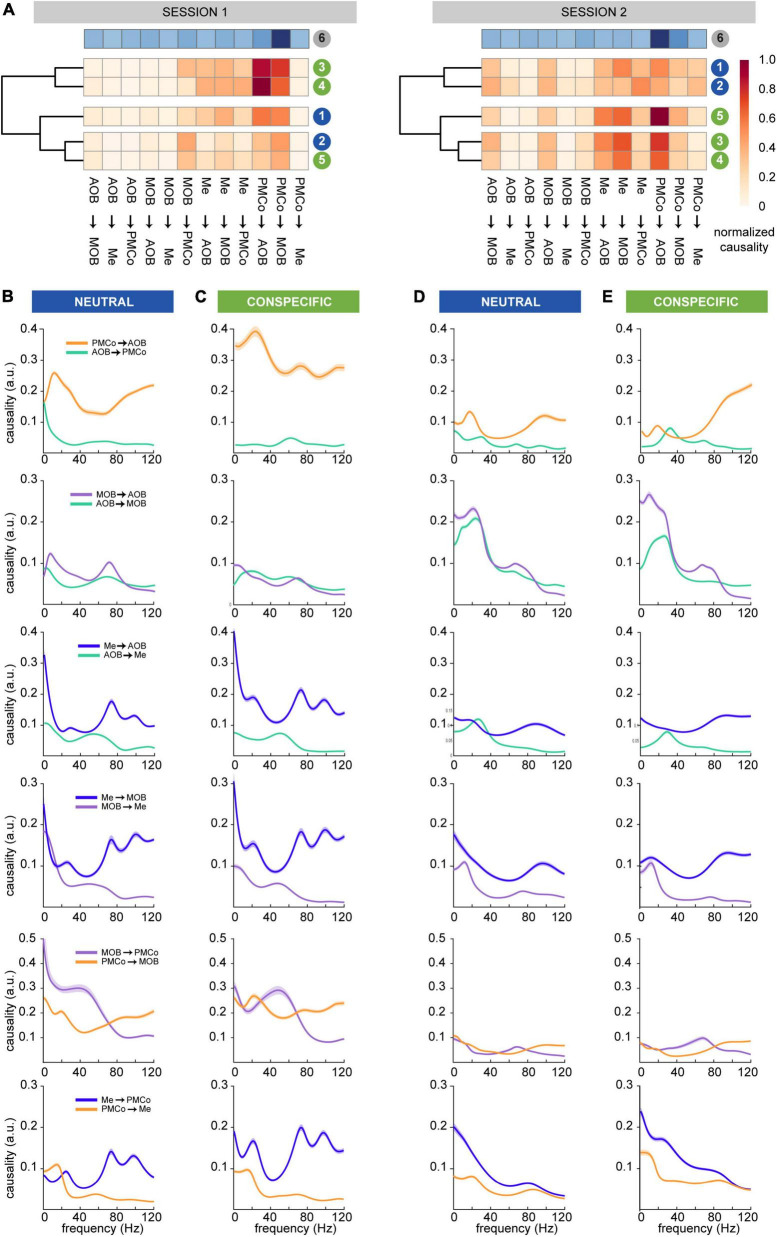
Granger’s causal analysis in the time and frequency domains. **(A)** Clustering of the similarity strength (arbitrary units) of the average causal values in the time domain for each stimulus for session 1 (left) and session 2 (right). The neutral stimuli, clean (1) and geraniol scented (2) bedding, in blue; and conspecific, castrated male (3), female (4) and intact male (5) soiled bedding, in green. In the top position, the pattern for non-exploration periods is represented in blues’ colormap, to allow comparison with the clustered patterns. **(B)** Averaged causal values in the frequency domain for selected pairs of nuclei during session 1 for neutral stimuli; **(C)** session 1 for conspecific stimuli; **(D)** session 2 for neutral stimuli; **(E)** session 2 for conspecific stimuli. **(B–E)** First row shows PMCo and AOB dynamics, second row MOB and AOB dynamics, third rows Me and AOB dynamics, fourth rows Me and MOB dynamics fifth row MOB and PMCo dunamics and sixth row Me and PMCo dynamics.

To highlight the common aspects of the causal activity for particular pairs of nuclei, we averaged the normalized causal values of all the exploration times for each stimulus and several non-exploration/resting times. An unsupervised classification method of the causal activity patterns was used to sort dissimilarities among the stimuli and led to defined clusters according to their Euclidean distance for both recording sessions (Figure 4A). In the first one, this analysis evidenced the presence of three clusters with a cophenetic correlation of 0.77, indicating that the clustering accurately reflects the original dissimilarity. Under these conditions, the neutral and conspecific stimuli patterns were intermingled between groups. The patterns that arose from castrated- and female-soiled bedding stimuli were clustered together (83% of the bootstrap iterations), whereas geraniol- and male-derived stimuli belonged to a second cluster (99%), with the clean-stimulus pattern as an isolated cluster (54%). Specifically, net information flows were detected from the amygdalar nuclei to bulbs, especially for female- and castrated-soiled bedding (cluster 3–4; Figure 4A, session 1).

Conversely, in the second session, the neutral and conspecific stimuli were observed in two different branches of the clustergram. In this case, neutral (bootstrap iteration, 86%) and conspecific (86.6%) stimuli were clustered in separate groups (cophenetic correlation = 0.65; Figure 4A, session 2). We could differentiate a solid cluster (92%) with castrated-male and female groups, distinguished from the male group.

These data suggest that the predominant causal profiles with conspecific stimuli are characterized by an amygdaloid control over the bulbs. In the particular case of the male-derived stimuli, the amygdaloid control appeared only in the second session. In contrast, the causal profile induced by neutral stimuli shows a sparser flow of information within the circuitry. These results suggest that the amygdala seems to have an active position in the different steps of chemosensory information processing, governing the activity of the entire network in the top-down sense. This suggestion is supported by the strong control of the amygdala over the bulbs during non-exploration/resting periods (Figure 4A, row 6).

The predominant causal flow PMCo→AOB in the frequency domain during the first session was remarkable in neutral and conspecific-derived stimuli for all frequency bands (Figures 4B,C). However, in the second session, the profiles changed, and significant causal peaks in PMCo→AOB direction shifted to a dominance of high-gamma frequencies (Figures 4D,E, PMCo→AOB); a novel beta-low gamma peak AOB→PMCo emerged for conspecific stimuli. The dynamics of the causality between the bulbs showed a rise in significant crosstalk between the AOB and MOB during the second session (Figure 4A, right). This change was particularly apparent for frequencies below 40 Hz, where the MOB dominated the causal stream, while for high-gamma frequencies the AOB drove the MOB (Figures 4D,E, MOB→AOB). The distribution of the peaks in the directionality from the Me to the bulbs was similar for neutral and conspecific stimuli in both sessions, which might reflect the bidirectional driving between the amygdala and the bulbs (Figures 4B–E, Me→MOB and Me→AOB). The causal flow between PMCo and MOB was very different in the first and second sessions. The first session showed a dominance under 60 Hz of the MOB for neutral stimuli and a peak around 50 Hz for conspecific stimuli (Figures 4B,C, MOB→PMCo); in the second session, this dominance is lighter (Figures 4D,E, MOB→PMCo). However, in the high-gamma frequencies, the PMCo dominates in all cases. The dynamics of the information flow between the amygdalar nuclei are different for each session but always dominated by the Me, especially in the first session (Figures 4B–E, Me→PMCo).

In summary, while studying the causal interactions of the bulbo-amygdalar network, both directionalities are recognized. The different patterns demonstrate a predominant functional state in which the amygdalar nuclei dominate the causal interaction.

### Theta-nested high frequencies define the bulbo–amygdalar neuronal processing

Neural oscillations arise from the temporal coordination of activity in organized networks of neurons (Buzsáki and Draguhn, 2004). The dynamics of these rhythms can thus reflect fast-paced changes in the local activity of neuronal assemblies during information processing. It is well-known that the theta rhythm (3–10 Hz) defines the chemoexploratory behavior in various brain structures and elicits a synchronic activity in the olfactory bulbs characterized by dominant theta rhythmicity with specific gamma coupling (Pardo-Bellver et al., 2017). This bimodal pattern becomes an ideal feature to shape the oscillatory organization of the network in response to particular inputs. The previous causal analysis has shown how the information flow in the olfactory-vomeronasal network is represented by various oscillatory profiles distributed in a wide range of frequencies. For this reason, we studied the theta-nested profile to differentiate states of activity involved in processing chemosensory stimuli.

**FIGURE 5:**
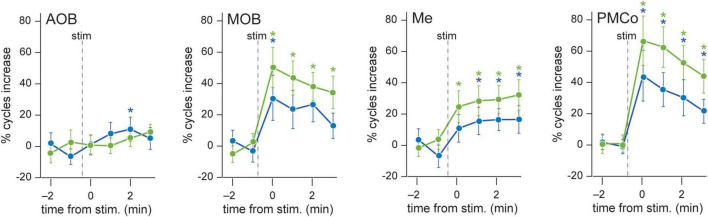
Evolution of theta-nested components upon stimulus presentation. Proportion of theta cycles coupled to nested components per minute, before and after neutral (blue) or conspecific (green) stimuli. Wilcoxon test comparing the basal against post-stimulus values, *p < 0.05.

**FIGURE 6:**
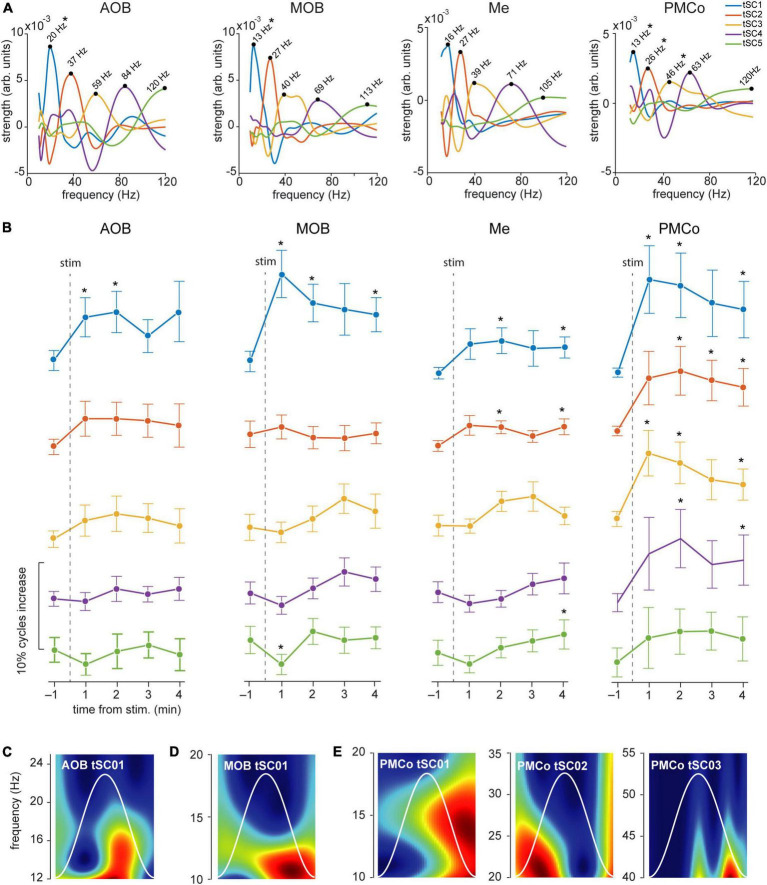
Temporal analysis of the theta cycles and evoked tSCs for neutral stimuli. **(A)** Spectral signatures in the frequency domain of the analyzed tSCs, for the four brain regions, during the exploration of neutral stimuli. Asterisks indicate those tSCs that showed a significant increase from the control period. **(B)** Proportion of theta cycles with nested components per minute of the five tSC, before and after the neutral stimuli presentation *p < 0.05 compared to pre-stimulus value. **(C)** Representative wavelet spectrogram of tSC1 in AOB; **(D)** tSC1 in MOB; **(E)** tSC1, tSC2, and tSC3 in PMCo.

**FIGURE 7:**
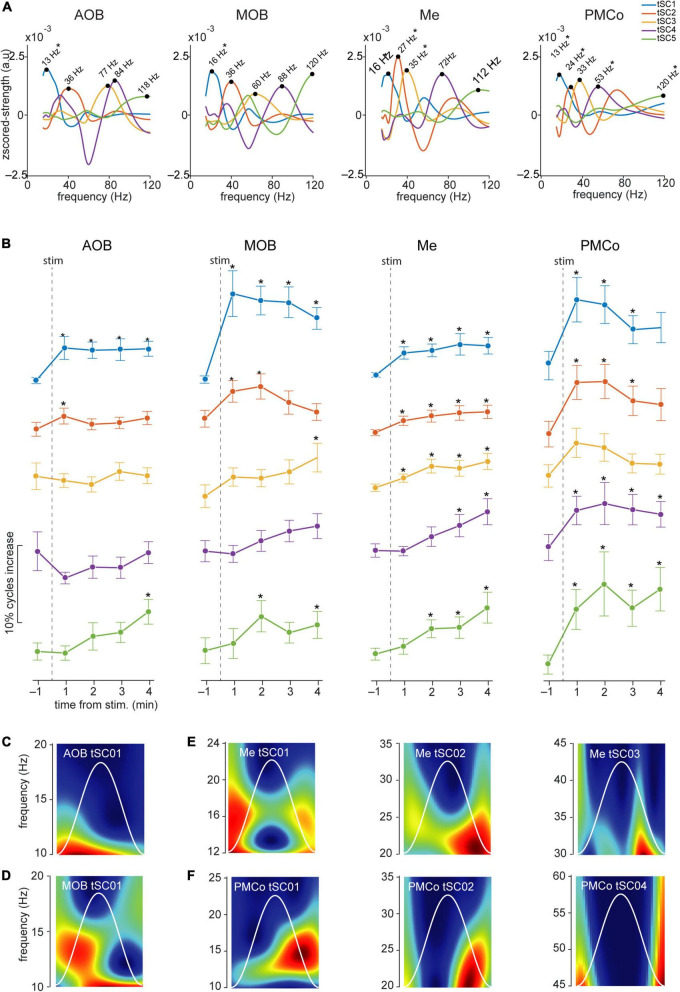
Temporal analysis of the theta cycles and evoked tSCs for conspecific stimuli. **(A)** Spectral signatures in the frequency domain of the analyzed tSCs, for the four brain regions during the exploration of conspecific-derived stimuli. Asterisks indicate those tSCs that showed a significant increase from the control period. **(B)** Proportion of theta cycles with nested components per minute of the five tSC, before and after the conspecific stimuli presentation *p < 0.05 compared to pre-stimulus value. **(C)** Representative wavelet spectrogram of tSC1 in AOB; **(D)** tSC1 in MOB; **(E)** tSC1, tSC2, and tSC3 in Me; **(F)** tSC1, tSC2, and tSC4 in PMCo.

First, we extracted the theta cycles (3–10 Hz) with coupled beta/gamma components before and during the exposure to the stimuli. The presence of neutral stimuli triggered a significant increase in the number of theta cycles with nested high-frequency components in the MOB, but not in the AOB (Figure 5 and Supplementary Table 1). As theta-nested spectral components (tSC) are thought to reflect coupling among and within brain areas (Lopes-Dos-Santos et al., 2018; Navas-Olive et al., 2020; Villafranca-Faus et al., 2021), we further investigated the spectral nature of the theta-coupled frequencies. As in previous works, we detected five significant tSCs across areas and stimuli. The lowest frequencies coupled to theta waves were observed in the beta band (10–30 Hz, Figures 6A, 7A), whereas most theta cycles showed coupling to gamma frequencies (30–120 Hz). It is worth remarking that the frequency peaks of the different tSCs do not strictly match among areas. Whereas the amygdalar tSC1 and tSC2 fall in the beta range, the bulb’s recordings showed tSC1 and tSC2 with close frequency peaks but falling in beta and gamma bands, respectively.

A detailed analysis of each tSC showed that the exploration of neutral stimuli (clean bedding and geraniol) led to an increase of theta cycles with nested beta coupling (12–20 Hz, tSC1) in both bulbs (Figures 6A,B, AOB and MOB; Figures 6C,D and Supplementary Table 2). Conversely, a noteworthy gain in the number of theta-nested high-frequency cycles was observed in the amygdaloid complex (Figure 5, Me and PMCo). This increase reflects the rise of tSC1-2 in the Me (Figure 6B and Supplementary Table 2) and tSC1 (∼13 Hz), tSC2 (∼27 Hz), and tSC3 (∼46 Hz) in the PMCo (Figures 6B,E and Supplementary Table 2), corresponding to beta and gamma oscillations (Figure 6A).

When soiled bedding from different conspecifics was present, the proportion of theta cycles with nested high frequencies increased in the MOB and both amygdalar nuclei (Figure 5 and Supplementary Table 1). The analysis of the specific tSC showed a significant increase in nested beta oscillations (∼13 Hz, tSC1) in the AOB (Figures 7A–C and Supplementary Table 3), and a transitory increase in the nested low-gamma waves (∼36 Hz, tSC2). Likewise, in the MOB there was a significant increase in the nested beta (∼16 Hz, tSC1, Figures 7A,B,D and Supplementary Table 3) and a transitory gain in the low-gamma (∼36 Hz, tSC2, Figures 7A,B). In the amygdala, the exploration of conspecific stimuli generates a significant rise of the nested beta (∼16 Hz, tSC1; ∼27 Hz, tSC2), and low-gamma (∼35 Hz, tSC3) during all the exploration periods in the Me (Figures 7A,B,E and Supplementary Table 3); whereas tSC4 (∼72 Hz) and tSC5 (∼112 Hz) showed lesser upsurge at the beginning but significant, nonetheless, at the end of the exploration period (Figures 7A,B,E and Supplementary Table 3). As with the neutral stimuli, almost every tSC in the PMCo significantly increased during conspecific exploration, evoking a surge of spectral components in the beta range (∼13 Hz, tSC1; ∼25 Hz, tSC2) and middle and high-gamma (∼53 Hz, tSC4; ∼120 Hz, tSC5; Figures 7A,B,F and Supplementary Table 3). Interestingly, the tSC1 of MOB and PMCo have similar values for neutral stimuli (∼13 Hz); while for conspecific stimuli, similarities are observed between the tSC1 of AOB and PMCo (∼13 Hz) and MOB and Me (∼16 Hz).

These data suggest that the exploration of conspecific stimuli is not only linked to the presence of theta activity in most chemosensory processing areas but promotes the appearance of nested high-frequency components within the theta cycles. Moreover, the processing in the olfactory bulbs preserved beta waves as coupled activity, while the amygdala revealed mixed beta and gamma oscillations.

**FIGURE 8:**
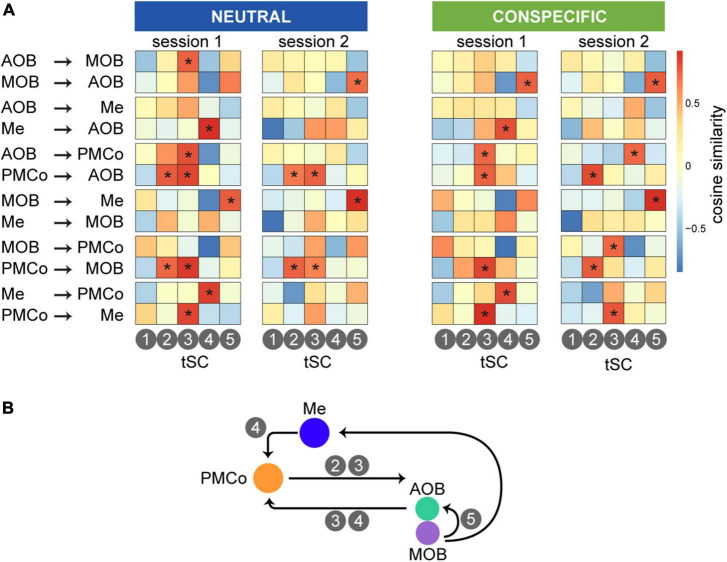
Causality-theta nested correlation for the set of grouped stimuli. **(A)** Similarity matrices ordered by causal interactions between areas (horizontal axis) and tSCs (vertical axis). In a color range between warms (reds, high similarity) and colds (blue, low similarity), we can observe how different theta components have a presence in the different causal directionality based on the similarity of both spectral profiles. Asterisks represent significance when the similarity values are above a threshold, defined as the 0.9 quantile of the normal distribution of the measures. **(B)** Schematic summary of the representative participation of the tSCs in the causal interaction.

Furthermore, we wanted to explore the possibility of relating the spectral causality profiles with the nested theta waves by a measure of similarity between both spectral distributions. Thus, we identified causal profiles (Figure 4) with a frequency distribution akin to the theta-nested components (Figures 6, 7). The results showed significant correlations of the tSCs with the spectral profiles of causality between interrelated areas, as summarized by the similarity map for each causal profile defined by source and target areas in Figure 8A. As a distinguishing feature, we observed high similarity values for the causal directionality from the amygdaloid areas (PMCo-Me) to the bulbs. Specifically, in both sessions, tSC2 and tSC3 are correlated with the causality explained in the PMCo-bulbs direction (Figure 8B). In a reversal direction, tSC3 and tSC4 showed high similarities with the spectral causality profiles between AOB and MOB, and PMCo. In turn, AOB and MOB exhibited causal profiles correlated with tSC5. Furthermore, the intraamygdalar activity showed a causal relationship between both nuclei in which the component tSC4 shows a high value of similarity.

Taken together, these data support the idea that complex neural activity in the bulbo–amygdaloid circuitry underlies the processing of information based on beta-gamma oscillations. The visualization of top-down control is also remarkable when we combine the causality data and the presence of theta-gamma coupling.

### The vomeronasal system is highly activated in female mice exposed to male urine in comparison to a neutral odorant

**FIGURE 9:**
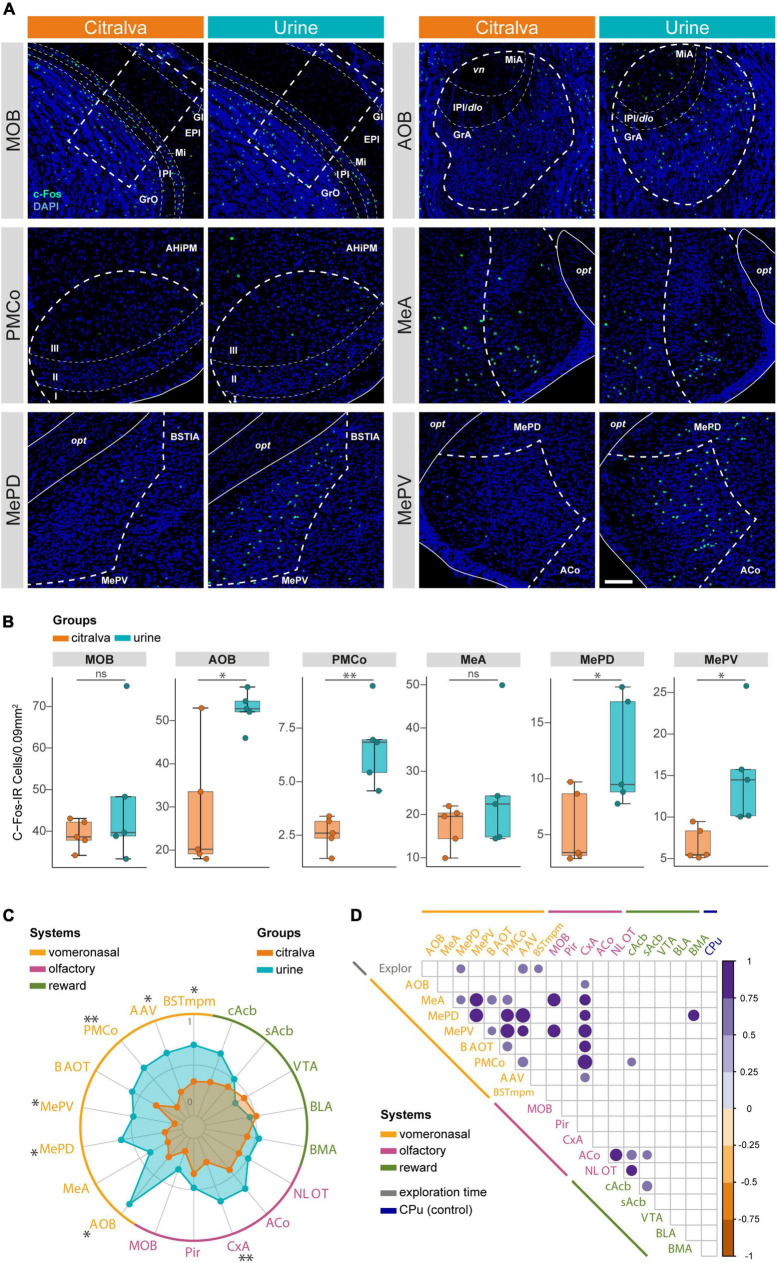
Global analysis of the immunohistochemical expression of c-Fos in the extended chemosensory network. **(A)** Confocal microscopy Z-stacks illustrating differential c-Fos expression between citralva (orange, *n* = 5) and urine (blue, *n* = 5) groups, exemplified by the most relevant structures of the bulbo-amygdalar network. Thick dashed lines indicate counting ROI, narrow dashed lines give further layering detail for some nuclei, whereas solid lines and surrounding structures provide neuroanatomical clues to facilitate area identification. Scale bar in MePD urine panel, valid for all images: 100 μm. **(B)** Group data distribution comparing cell activation density (c-Fos-IR cells/0.09 mm^2^) between citralva and urine groups in the nuclei displayed in **(A)**. Box plots are defined in terms of minima and maxima by whiskers, and the center and bounds of box by quartiles (Q1–Q3). Outliers were identified by Tukey method and left in the analysis. Two-sided *t* or Mann-Whitney *U* test: *p < 0.05; ^**^p < 0.01. Other comparisons are shown in Supplementary Figure 1. **(C)** Extended analysis of the pattern of c-Fos-IR density in the vomeronasal (yellow), olfactory (magenta), and reward (green) circuits of female mice exposed to citralva or male urine. Each axis of the radar plot represents a measured nucleus. The vertices defining the colored polygons represent the mean of the Min-Max normalized data expressed for each group, illustrating the pattern of activity in both experimental groups within each system. Statistically significant structures are indicated with asterisks. Two-sided *t* or Mann-Whitney *U* test: *p < 0.05; ^**^p < 0.01. **(D)** Correlogram displaying significant statistical correlations between pairs of variables, comparing altogether c-Fos cell density in all nuclei and exploratory behavior. Circle size and color represent Pearson’s correlation *r*-value and are only present when *p* < 0.05. Circle radius has been rescaled according to the minimum Pearson’s *r*-value which *p*-value was significant. AOB, accessory olfactory bulb; MeA, anterior medial amygdaloid nucleus; MePD, posterodorsal medial amygdaloid nucleus; MePV, posteroventral medial amygdaloid nucleus; BAOT, bed nucleus of the accessory olfactory tract; PMCo, posteromedial cortical amygdaloid nucleus; AAV, anterior amygdaloid area, ventral part; BSTmpm, medial posteromedial bed nucleus of the stria terminalis; MOB, main olfactory bulb; Pir, piriform cortex; CxA, cortex amygdala transition area; ACo, anterior cortical amygdaloid nucleus; NLOT, nucleus of the lateral olfactory tract; cAcb, nucleus accumbens core; sAcb, nucleus accumbens shell; VTA, ventral tegmental area; BLA, basolateral amygdaloid nucleus; BMA, basomedial amygdaloid nucleus; CPu, caudate putamen (striatum); Explor, exploration time; GrO, granule cell layer of the MOB; IPl, internal plexiform layer of the MOB; Mi, mitral cell layer of the MOB; EPl, external plexiform layer of the MOB; Gl, glomerular layer of the MOB; GrA, granule cell layer of the AOB; IPl/dlo, internal plexiform layer of the AOB/dorsal lateral olfactory tract; MiA, mitral cell layer of the AOB; vn, vomeronasal nerve; AHiPM, posteromedial amygdalohippocampal area; opt, optic tract; BSTIA, intraamygdaloid bed nucleus of the stria terminalis; IR, immunoreactive.

To evaluate brain activity to a broader extent, we analyzed c-Fos expression in several related structures in female mice exposed to either male urine, as a conspecific stimulus, or citralva, as a neutral stimulus. At the expense of temporal resolution, c-Fos immunohistochemistry (Figure 9A) allowed us to study brain activation in a widespread network of distributed brain regions, including structures from the vomeronasal, olfactory, and reward systems. As reported, animals exposed to bedding with male urine exhibited a significantly longer exploration time than those exposed to citralva-scented bedding (Villafranca-Faus et al., 2021).

In addition to the vomeronasal structures already considered in the electrophysiological experiment (AOB, PMCo, and Me), we included the medial posteromedial bed nucleus of the *stria terminalis* (BSTmpm), the ventral part of the anterior amygdaloid area (AAV) and the bed nucleus of the *accessory olfactory tract* (BAOT; Gutiérrez-Castellanos et al., 2010). As expected, the expression of c-Fos in the AOB was significantly higher in the conspecific group (two-sided *t*-test, *t*_8_ = 3.44; *p* = 0.021; Figures 9B,C). In the same way, the PMCo was also more active when the females were exposed to male urine (*t*_8_ = 4.51; *p* = 0.005; Figures 9B,C). This approach allowed us to differentiate among the Me functional subdivisions (MeA, anterior Me; MePD, posterodorsal Me; MePV, posteroventral Me; Choi et al., 2005; Pardo-Bellver et al., 2012). Within the medial amygdala, significant differences were found in both the MePD (*t*_8_ = 2.51; *p* = 0.04) and MePV (*t*_8_ = 2.82; *p* = 0.039) subdivisions, whereas the MeA portion exhibited no differences (Mann-Whitney *U* test, *U* = 6; *p* = 0.222; Figures 9B,C). In the rest of the vomeronasal system, the comparison of c-Fos-IR cell density revealed higher activation in the BSTmpm (*t*_8_ = 2.51; *p* = 0.038) and the AAV (*t*_8_ = 2.35; *p* = 0.0498) in the conspecific group (Figure 9C and Supplementary Figure 2). By contrast, no significant increment of c-Fos expression was found in the BAOT (*t*_8_ = 1.34; *p* = 0.217; Figure 9C and Supplementary Figure 2).

To examine the possible effects induced by urine volatiles or the potential indirect activation that might be due to non-volatile compounds, we assessed the neuronal activation of the MOB, the piriform cortex (Pir) and the main structures of the olfactory amygdala, namely the nucleus of the *lateral olfactory tract* (NLOT), the anterior cortical nucleus (ACo), and the cortex-amygdala transition area (CxA). Compared to the c-Fos expression observed in the group exposed to citralva, the exploration of male urine had no effect in the MOB (t_8_ = 1.03; *p* = 0.353; Figures 9B,C), the Pir (t_8_ = 0.77; *p* = 0.478), the ACo (t_8_ = 1.22; *p* = 0.261), or the NLOT (*t*_8_ = 0.48; *p* = 0.647; Figure 9C and Supplementary Figure 2). Surprisingly, the CxA showed a strong effect of the urine exposure (*U* = 0; *p* = 0.008; Figure 9C and Supplementary Figure 2).

Since the urine of conspecific males induced a preferential exploration in the females, we looked for c-Fos expression changes in the reward system, including the tegmento-striatal and amygdalo-striatal circuits. However, no significant differences were observed either in the mesolimbic pathway (ventral tegmental area: *t*_8_ = 0.40; *p* = 0.701; accumbens core: *t*_8_ = 1.39; *p* = 0.202; accumbens medial shell: *t*_8_ = 0.49; *p* = 0.646) or in the basolateral complex of the amygdala (basolateral: *t*_8_ = 0.28; *p* = 0.786; basomedial: *t*_8_ = 0.50; *p* = 0.632; Figure 9C and Supplementary Figure 2).

To exclude possible differences in the histological processing or other non-controlled factors in the previous comparisons, we analyzed the c-Fos expression in the caudate-putamen as a non-chemosensory related area. The exposure to male urine had no effect in the activity of the caudate-putamen (t_8_ = 0.02; *p* = 0.982; Supplementary Figure 2) when compared to citralva, thereby providing a proper control measurement.

Comparative analysis of the neuronal activation elicited by male urine or a neutral odorant in females indicates that the vomeronasal system, but not the olfactory (except for the CxA) or reward systems, is more activated when exposed to male urine.

### Correlation analysis suggests a similar activation pattern in the whole vomeronasal system

To explore whether the activity pattern in the olfactory, vomeronasal, and reward systems are related to each other, we analyzed statistical correlations among the c-Fos density in the different nuclei. In addition, we also analyzed the correlation of c-Fos expression (present results) with the time spent exploring the stimuli (reported in Villafranca-Faus et al., 2021). Correlations were assessed by pooling together all subjects, independent of the experimental group.

Several significant correlations were found (Figure 9D and Supplementary Table 4). Multiple positive correlations were evidenced within the vomeronasal system, indicating that many vomeronasal nuclei had similar c-Fos expression pattern in response to the stimuli. Specifically, in the medial amygdala strong positive correlations were found between the MePV and the MePD (*r* = 0.88; *p* = 0.001) and MeA (*r* = 0.87; *p* = 0.001), and between the MePD and the MeA (*r* = 0.66; *p* = 0.038), thus asserting that all three subdivisions of the medial amygdala responded in a similar way to the cues. Furthermore, c-Fos cell density in PMCo presented robust correlations with the functional portions of the medial amygdala: MePD (*r* = 0.85; *p* = 0.002), MePV (*r* = 0.89; *p* = 0.0004), and MeA (*r* = 0.71; *p* = 0.023). Besides this, the PMCo also positively correlates with the BAOT (*r* = 0.70; *p* = 0.025) and the AAV (*r* = 0.73; *p* = 0.016), and shows a trend with the AOB (*r* = 0.62; *p* = 0.055). In addition, BAOT neuronal activation displayed direct correlation with MePV (*r* = 0.64; *p* = 0.046) and MeA (*r* = 0.69; *p* = 0.028), whereas AAV presented high-positive correlation with MePD (*r* = 0.93; *p* = 0.0001) and MePV (*r* = 0.76; *p* = 0.011).

It is worth mentioning that the CxA c-Fos density correlated with the activation of almost all vomeronasal nuclei, corroborating its importance in the processing of conspecific signals, as shown in the previous section. Significant positive correlations were found between the CxA and the AOB (*r* = 0.64; *p* = 0.047), MeA (*r* = 0.77; *p* = 0.009), MePD (*r* = 0.75; *p* = 0.012), MePV (*r* = 0.86; *p* = 0.001), BAOT (*r* = 0.79; *p* = 0.006), PMCo (*r* = 0.93; *p* = 0.0001), and AAV (*r* = 0.68; *p* = 0.030).

Relevant correlations between the MOB and other structures of the accessory olfactory system were present: MeA (*r* = 0.86; *p* = 0.002), MePV (*r* = 0.85; *p* = 0.002), and a trend with the PMCo (*r* = 0.6; *p* = 0.067). Surprisingly, no correlation was observed between the MOB and the Pir (*r* = 0.03; *p* = 0.945).

With regard to the behavioral outcome, we found direct correlation between the time exploring the stimuli and the activation of some vomeronasal structures, such as the MePD (*r* = 0.66; *p* = 0.040), the AAV (*r* = 0.70; *p* = 0.025), and the BSTmpm (*r* = 0.64; *p* = 0.046).

Among the structures of the olfactory amygdala, leaving apart the CxA, significant correlations appeared only between the activity in the ACo and the NLOT (*r* = 0.82; *p* = 0.003). Finally the activity in the chemosensory systems showed very scarce correlations with the structures in the reward system. The activity in the accumbens core showed significant correlation with that in the PMCo (*r* = 0.65; *p* = 0.042), ACo (*r* = 0.72; *p* = 0.019), and NLOT (*r* = 0.75; *p* = 0.012). In addition, the c-Fos density in ACo also correlated with that in the accumbens shell (*r* = 0.68; *p* = 0.031). As expected, no correlation was found between the caudate-putamen and any other structure, thus confirming its validity as a control.

### Principal component analysis reveals a distinctive c-Fos expression pattern in the chemosensory and reward circuits

**FIGURE 10:**
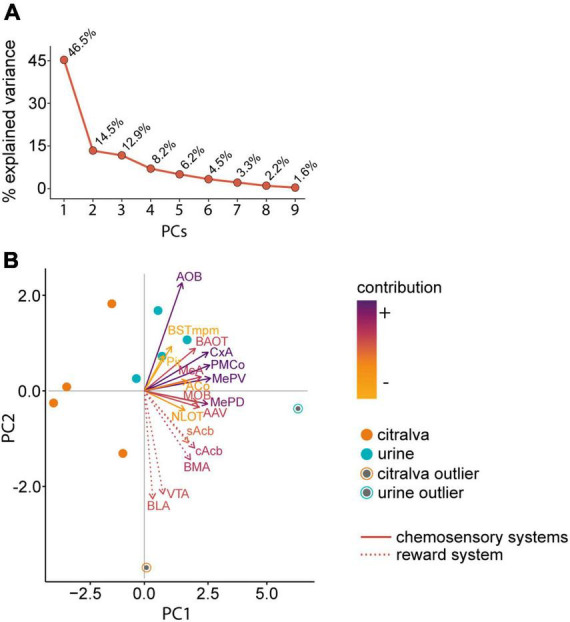
Principal component analysis of the c-Fos cell density data in the chemosensory and reward systems. **(A)** Percentage of explained variance by each principal component. **(B)** PCA biplot showing females exposed to male urine (blue dots, *n* = 5) or citralva (orange dots, *n* = 5). Dots represent the loading of each animal to the PCs. Outliers, represented as black dots, were identified by the Mahalanobis distance method and left in the analysis. Solid vector lines represent chemosensory structures and dashed vector lines reward system nuclei. Darker color of the vector implies more contribution, whereas lighter color implies less contribution. The orientation of the vector indicates how much the variable contributes to the PC: the more parallel to a principal component axis, the more it contributes only to that PC. The length of the vector indicates how well the two principal components explain the variability of the density of c-Fos expression for that nucleus. The angles between vectors corresponding to different nuclei show their correlation: small angles represent high positive correlation, right angles represent lack of correlation, opposite angles represent high negative correlation. AOB, accessory olfactory bulb; MeA, anterior medial amygdaloid nucleus; MePD, posterodorsal medial amygdaloid nucleus; MePV, posteroventral medial amygdaloid nucleus; BAOT, bed nucleus of the accessory olfactory tract; PMCo, posteromedial cortical amygdaloid nucleus; AAV, anterior amygdaloid area ventral part; BSTmpm, medial posteromedial bed nucleus of the stria terminalis; MOB, main olfactory bulb; Pir, piriform cortex; CxA, cortex amygdala transition area; ACo, anterior cortical amygdaloid nucleus; NLOT, nucleus of the lateral olfactory tract; cAcb, nucleus accumbens core; sAcb, nucleus accumbens shell; VTA, ventral tegmental area; BLA, basolateral amygdaloid nucleus; BMA, basomedial amygdaloid nucleus; PC, principal component.

To identify global activity patterns and thus interconnected neural response networks, we performed a PCA of the c-Fos data. In addition, this approach revealed which structures contribute the most to our model. We selected the first two out of the nine PCs obtained, as they clearly separated both experimental groups and explained 61.0% of the total variance (PC1 46.5% and PC2 14.5%; Figure 10A).

As shown in the biplot (Figure 10B), females exposed to male urine clustered around the vectors of the chemosensory nuclei, whereas females exposed to citralva appear randomly distributed. For the PC1, the loading factors only presented positive values (*X*-axis; Figure 10B). This component stood for a weighted average of the whole network activity; therefore, it did not allow us to discriminate between functional systems. By contrast, the PC2 had both positive and negative loading factors (*Y*-axis; Figure 10B). For this component, the vectors generated from the variables of the reward system presented the most negative values in the distribution, whereas the vectors originated by the chemosensory nuclei had, in all cases, higher values. This separation indicates that the set of nuclei analyzed belong to two particular functional systems that respond differently to the stimuli. Concerning the chemosensory network, PC2 values did not allow to discrimination between the olfactory and vomeronasal systems.

The variables that showed a higher contribution (weighted sum of the absolute value of loading to each component) to the model were the AOB (0.15), PMCo (0.17), MePD (0.15), MePV (0.16), and CxA (0.17). Particularly, the variable that presented the highest contribution to the PC1 was the MePV (10.6%), whereas the one that contributed the most to the PC2 was the AOB (21.9%). In this regard, PCA matches the previous results from independent data comparisons and correlations, providing further understanding of the relevance of these nuclei in chemosignal processing.

## Discussion

### Top-down and bottom-up information transfer between the amygdala and the main and accessory olfactory bulbs

The present results reveal that exploring neutral odorants or conspecific chemosignals in mice simultaneously elicits the neuronal activation of both olfactory neuronal circuitries, originating in the main and accessory olfactory bulbs. Thus, according to the initial hypothesis, processing chemosensory stimuli requires both olfactory and vomeronasal structures, as revealed by the causal patterns of activity among the MOB, AOB, and vomeronasal amygdala, as well as the correlation observed in the c-Fos activity between the structures of the vomeronasal system and the CxA and, to a lesser extent, with the MOB. The information derived from chemical signals is integrated into complex neural processing capable of generating a complete picture of the chemicals present in the environment (Baum and Kelliher, 2009; Martínez-García et al., 2009). Active detection during sniffing entails a rhythmic pattern coupled to breathing in which odors enter into direct contact with the olfactory receptors (Manabe and Mori, 2013). With the incorporation of airflow into the nasal cavity, a second path directs non-volatile chemicals into the vomeronasal organ (Meredith and O’Connell, 1979), where the receptors are located. Thus, the main and accessory olfactory systems rely on two mechanisms for internalizing chemical stimuli, which appear to operate synchronously and influence the neural processing of the chemosensory information. Consequently, the neural activities in both systems remain coupled (Pardo-Bellver et al., 2017).

**FIGURE 11:**
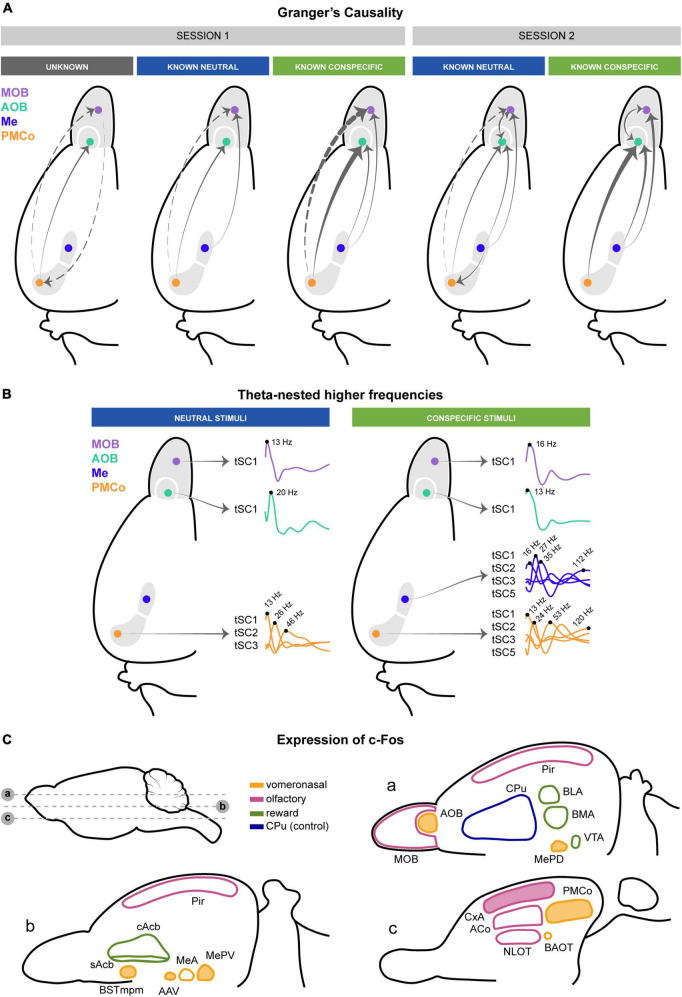
Schematic summary of the causality, theta-nested higher frequencies, and c-Fos expression involved in processing chemosensory cues. **(A)** Top-down and bottom-up transfer of information between the amygdala and the main and accessory olfactory bulbs summarizing Figure 3A. The thickness of the arrows reflects the intensity of the GC. Dashed arrows indicate indirect anatomical connections. **(B)** Theta-nested higher frequencies define the bulbo–amygdalar neuronal processing. Only tSCs with significant increases are shown. **(C)** Activation of the vomeronasal systems and CxA by male-derived chemical signals revealed by c-Fos expression. Significant (filled) and non-significant (non-filled) structures in the vomeronasal (yellow), olfactory (magenta), and reward (green) circuits of female mice exposed to citralva or male urine. The caudatus-putament, analyzed as control, is depicted in blue.

In our analysis, we have identified motifs of causal interaction among the primary nodes of the bulbo–amygdalar network in the presence of a set of biologically salient stimuli. We found a different clustering of these motifs that seems to depend on the animal’s experience to the stimuli; thus, the causal analysis indicates a different clustering for the two experimental sessions (Figure 11A). In this scenario, the clean stimulus would reflect the basal activation of the chemosensory network, as it is the vehicle for the rest of the stimuli. In session 1, the cluster comprising female and castrated-male stimuli could represent the causal flow generated by known conspecific stimuli (Figure 11A, session 1, conspecific stimuli). This could be explained by the absence of testosterone-dependent signals in the castrated-male urine, which may be recognized as the chemical pattern of juvenile male littermates (Penn et al., 2022). However, geraniol and intact males appear together in the clustergram, possibly due to the lack of prior experience with them (Figure 11A, session 1, unknown). After several re-exposures to these chemicals, during session 2, we can see how neutral and conspecific stimuli are segregated (Figure 11A, session2). Conspecific cues trigger high causal values from the amygdalar nuclei to the bulbs, especially in intact male stimuli, leading to its separation from the others. Consequently, the causal dominance shift after re-exposure to male cues suggests that the vomeronasal amygdala might direct the process of conspecific identification. Consistent with the changes in the clustering analysis observed from session 1 to session 2, differences in exploratory behavior across sessions are also observed. On the one hand, there is a global effect on the exploration time, which is lower in session 2. However, this effect is only significant for geraniol, indicating a lack of habituation to the conspecific stimuli. Intra-session habituation is also present only for geraniol.

A relevant finding has been the substantial top-down control that the vomeronasal amygdala (Me and PMCo) exerts over the activity of the olfactory bulbs, which is certainly consistent during active exploration, especially in response to conspecific urines. Indeed, the causal values have evidenced bidirectionality between both areas. In epochs of chemosignal exploration, the top-down control from the amygdala over the bulbs alternates with brief periods in which this control weakens and a light bottom-up flow of information from the bulbs reaches the vomeronasal amygdala. However, even in these “input periods,” the transit of neural information is still more robust in the direction from the amygdala to bulbs.

This view is supported by the anatomy of the connections between structures. The PMCo and the MePV give rise to dense projections to the granular layer of the AOB (Pardo-Bellver et al., 2012; Gutiérrez-Castellanos et al., 2014), and in turn, the MeA projects directly to the mitral cell layer of the AOB (Pardo-Bellver et al., 2012). Thus, the top-down control of the vomeronasal amygdala over the AOB has a clear substrate in these centrifugal projections. The PMCo and MePV projections to the granular layer of the AOB are glutamatergic (Fan and Luo, 2009), and innervate the GABAergic granular cells providing intrabulbar inhibition. The GABAergic activity of the granular cells in the MOB has been described as the source of the gamma rhythm (Lepousez and Lledo, 2013), and probably the same substrate could explain the presence of gamma bursts in the AOB, as we have observed in our results. Hence, the glutamatergic control of the amygdala over the inhibitory granule cells in the AOB might be the underlying substrate of the causality in the high-gamma range observed in the present results.

Similarly, GC also reveals an amygdaloid control on the activity of the MOB. In this case, there are no direct projections from the vomeronasal amygdala to the MOB; thus, the circuitry that mediates this functional relation should include relay structures. A major candidate is the piriform cortex, which receives projections from the PMCo (Gutiérrez-Castellanos et al., 2014) and, to a lesser extent, also from the Me (Pardo-Bellver et al., 2012). Moreover, the olfactory cortex gives rise to a dense projection of the MOB granular layer (Martínez-García et al., 2009). Structures of the olfactory amygdala, such as the ACo, CxA, NLOT, and the posterolateral cortical amygdaloid nucleus, are interconnected with the Me and PMCo (Santiago and Shammah-Lagnado, 2004; Cádiz-Moretti et al., 2016a,^2017^) and also project to the MOB, although quite sparsely (de Olmos et al., 1978; Mohedano-Moriano et al., 2012). Among these structures, the c-Fos results show that the CxA stands out as an interface between the olfactory and vomeronasal amygdaloid divisions (see below).

The causality analysis in the frequency domain shows that different oscillatory profiles support the top-down and bottom-up streams. While the downward control occurs in the range of high-gamma oscillations, the upward flow implies activity at beta and low gamma frequencies (20–40 Hz). The oscillations in the beta range were not analyzed in our previous work (Pardo-Bellver et al., 2017), but the present results clearly indicate that they are very likely key actors in the transfer of information from the bulbs to the amygdala. A similar observation, also derived from the GC analysis, has been reported for the indirect input from the MOB to the hippocampus (Lockmann et al., 2018). Thus, oscillatory activity in the beta-low gamma range seems crucial to transferring information from the olfactory bulbs to the amygdala and the hippocampus. Numerous studies have previously described that odorant stimulation shifts the MOB from high-gamma to beta activity (Martin and Ravel, 2014). Our results suggest that this transition in oscillatory activity also occurs in the AOB and is related to a change in the flow of information from the top-down to the bottom-up direction. This is not to say that the gamma activity in the bulbs is generated by the centrifugal input, since prior evidence indicates that it originates locally as a result of the reciprocal synapses between the granule and mitral cells (Kay et al., 2009; Lepousez and Lledo, 2013). However, in terms of this information transit inferred from the GC analysis, the strong influence of the amygdaloid centrifugal input to the AOB and MOB is observed in the high-gamma band. In the same way, several previous reports indicate that the beta rhythm in the MOB requires centrifugal input from the piriform cortex (Martin et al., 2004, 2006). Our results do not contradict these observations since the GC does not show the origin of the oscillations but the directionality of the information flow.

Our data show a bidirectional communication between both bulbs in session 2 following re-exposure to the stimuli. Although there is evidence for a direct link from the AOB to the MOB (Vargas-Barroso et al., 2016), the fact that the crosstalk between the bulbs only appears in session 2, as reflected by GC analysis, suggests that the strong top-down control may also be directing this reciprocal communication.

We have shown that there is an alternating bidirectional transmission of the neural activity from the areas responsible for the sensory acquisition of stimuli (bulb circuitry) and those concerned with encoding this particular class of incoming information (vomeronasal amygdala). This scheme suggests that sensory processing depends, in part, on the neural areas that have the competence to activate behavioral patterns. Consequently, the top-down control proposed in this work could maximize the efficiency of the sensory input procedure in an action-perception loop (Buzsáki, 2019).

The results derived from the GC analysis have shown intricate communication dynamics within the bulbo–amygdalar network dependent on the biological relevance of the stimulus. This complexity might be linked to changes in the neural activity represented by local oscillatory patterns of the network nodes. We analyzed the intra-nucleus theta nested high-frequency components associated with stimulus exploration. Our observations show that the number of theta cycles with significant nested components rises when a stimulus is present, indicating an increment in the local computations that shape the intra-nucleus processing of chemosensory cues. The single-cycle spectral components identified in this work exhibit frequencies consistent with previously reported activity in the MOB during active processing, especially beta, low and high-gamma oscillations (Lepousez and Lledo, 2013; Manabe and Mori, 2013; Rojas-Líbano et al., 2014), in the AOB (Binns and Brennan, 2005; Ben-Shaul et al., 2010; Tendler and Wagner, 2015; Pardo-Bellver et al., 2017) and amygdala (Binns and Brennan, 2005; Pardo-Bellver et al., 2017; John et al., 2022). Contrary to these previous works, where frequency bands were pre-established, the unsupervised tSC analysis determines oscillatory activity closer to the working frequencies of these nuclei, thus providing a more accurate description of the local computations.

The analysis of the high-frequency tSCs in the olfactory bulbs and the vomeronasal amygdala, extracted two components within the range of beta and low gamma frequencies (20–40 Hz) in which the GC values indicate the bottom-up directionality. Similar tSCs are also observed in the Me and PMCo. In addition, a high-gamma component (>100Hz) is present in all four structures, corresponding to the frequency range in which the top-down causality is observed. Although both neutral and conspecific-derived stimuli increase the local processing within the network, it is worth mentioning that conspecific-derived stimuli generate a broader diversity of nested components in the amygdalar complex (Figure 11B, conspecific stimuli), likely reflecting the biological complexity of these stimuli. This increase in theta-gamma cycles is consistent with the stronger top-down GC observed for conspecifics.

We have devised a method for detecting similarities between the spectral causal profiles and the representative theta-nested components. When we integrated theta-nested components in the causal data, we observed an influence of beta and gamma oscillations on the communication inside the bulbo–amygdalar network. These activity modes are correlated with a causal relationship between the neuronal elements of the network. We observe clear activity peaks in a wide range of frequencies in the spectral causal profiles. Although these activity peaks do not exactly match the causality peaks described for the top-down and bottom-up flows of information, it should be noted that the tSCs represent local processing within each structure, instead of transfer of information. In addition, the similarity between the spectral causal profiles and the tSCs should be interpreted as a correlation, not as a causal relation. In any case, our results indicate that individual tSCs can be correlated with causal profiles.

Interestingly, a leading role of certain tSCs can be related to specific causal relations among distinct areas. The predominance of activity flows from amygdalar nodes to bulbs correlates with specific tSCs, suggesting that the tSCs could be attributed to different encoding phases concerning the transfer of information into the network. Our results also point to a top-down control when we include high-oscillation patterns in the causal interaction among areas. Overall, our data suggest that the encoding of vomeronasal inputs, represented locally with beta-gamma neuronal activity, is integrated into a more general flow of information in the amygdalo–bulbar circuit.

### The vomeronasal system and the CxA are activated by male-derived chemical signals

Assessment of c-Fos expression in the chemosensory and reward systems, after the exposure to male-derived signals or a neutral odorant, revealed a distinct neural activity pattern specifically in the vomeronasal system of female mice (Figure 11C). This finding corroborates the well-established idea that non-volatile signals in male mice urine stimulate the vomeronasal system (Moncho-Bogani et al., 2005). Previous works, however, did not use animals exposed to a neutral odorant as controls (Halem et al., 1999; Moncho-Bogani et al., 2005). This experimental design allowed us to distinguish between general activity induced by a neutral odor exposure and activity elicited specifically by male chemical signals.

Among the vomeronasal system, no significant increment of c-Fos density was found in the MeA and the BAOT. The MeA receives important projections from the MOB, whereas the olfactory input to the posterior subdivisions of the Me is much more limited (Scalia and Winans, 1975; Lehman and Winans, 1982; Wood and Coolen, 1997; Cádiz-Moretti et al., 2016b). Therefore, MeA could be activated by the exposure to citralva. Furthermore, the MeA acts as a chemosensory filter that allows discrimination between same-sex or opposite-sex conspecifics (Maras and Petrulis, 2010). In these terms, it should be noted that the behavioral task we performed did not require discrimination between different stimuli, but a single initial activation of the nucleus. Regarding the BAOT, it receives a clearly preponderant innervation from the AOB (Gutiérrez-Castellanos et al., 2010). The lack of differences, in this case, may be due to methodological limitation in c-Fos quantification, resulting from the small size of the nucleus. Notwithstanding this, multiple positive correlations were evidenced within the vomeronasal nuclei, suggesting a comparable activation pattern of the whole vomeronasal system in response to chemical cues.

Unexpectedly, CxA activity correlated positively with many of these vomeronasal nuclei and presented a robust response to male urine. Although this structure is predominantly olfactory, it also receives a sparse direct innervation from the AOB (Gutiérrez-Castellanos et al., 2010). The neurochemistry and connectivity of this area suggest that it might be involved in the processing of olfactory information with relevant biological meaning, such as male-derived volatiles (Cádiz-Moretti et al., 2016a). Our results are consistent with this hypothesis and point to the CxA as an important interface between the olfactory and vomeronasal amygdaloid structures.

The positive correlations of the c-Fos expression between the Me and MOB, and the trend of PMCo with both bulbs, may be related to the bidirectional relation between the amygdala and the main and accessory olfactory bulbs observed in the GC analysis. Since the activity in the olfactory bulbs is not correlated with the time spent exploring the chemosensory stimuli, these correlations may reflect the amygdaloid control of the bulbar activity instead of the input of sensory information.

Male-derived pheromones in the urine are attractive for females (Roberts et al., 2010), which leads to a preference that is reflected in an increased exploration time when compared to a neutral odorant (Villafranca-Faus et al., 2021). However, the current results show no significant c-Fos expression differences in the mesolimbic pathway or the basolateral complex of the amygdala (Figure 11C). As reported in Moncho-Bogani et al. (2005), c-Fos cell density in the reward system increases in response to male-soiled bedding if compared to clean bedding. This finding suggests that citralva might have some hedonic value as shown for the similar molecule citronellol (Midroit et al., 2021), thus activating the reward system, despite being an odorant with no relevant biological meaning. Additionally, neurotoxic lesions of the dopaminergic mesolimbic pathway do not impair female preference for male-derived pheromones (Martínez-Hernández et al., 2012), although again in this work the controls were exposed to clean bedding. Therefore, more research is needed to clarify the circuits mediating the observed preference.

To further understand the global pattern of neural activity and identify particular functional networks that responded differently to the stimuli, we performed a PCA. Since our analysis included a large set of variables, this method enabled us to reduce overall noise in the model. The PCA results reveal distinctive c-Fos expression patterns in the chemosensory and reward circuits. However, no discrimination appears between the vomeronasal and olfactory systems. This result might be explained by the need of convergence of strict olfactory and pheromonal information (Baum and Kelliher, 2009; Keller et al., 2009), characterized here as similar c-Fos expression patterns among the vomeronasal and olfactory nuclei, according to the PCA. Strikingly, the nuclei targeted in the electrophysiological experiment (AOB, PMCo, and Me) and the CxA, contribute the highest to the PCA model, corroborating their relevance in chemosensory information encoding.

In summary, the analysis of the activity patterns in the olfactory bulbs and the vomeronasal amygdala shows that even neutral olfactory stimuli induce the transfer of information between the main and accessory olfactory systems, not only in the direction of the sensory input but, especially, in the top-down control of the amygdala over the bulbs. The c-Fos analysis supports this interaction between the two chemosensory systems and reveals that the cortex–amygdala transition area is probably a key player in integrating olfactory and vomeronasal information in the amygdala.

## Data availability statement

The raw data supporting the conclusions of this article will be made available by the authors, without undue reservation.

## Ethics statement

The animal study was reviewed and approved by Research Ethics and Animal Welfare Committee of the University of Valencia.

## Author contributions

CP-B, MV-M, EL, and VT-M designed the study. CP-B and SM-B performed the electrophysiological experiments. MV-M and AT-S performed the c-Fos experiments. SM-B, CP-B, MV-M, MV-F, CS-B, AT-S, SD, JM-R, AC-F, EL, and VT-M analyzed and interpreted the data. CP-B, SM-B, MV-M, AT-S, CS-B, JM-R, AC-F, FM-G, VT-M, and EL prepared the manuscript draft. EL and VT-M supervised the study and obtained funding. All authors revised, modified, and approved the final manuscript.
