# Supplementary material for: Neural activity patterns in the chemosensory network encoding vomeronasal and olfactory information in mice
Source: Front Neuroanat. 2022 Sep 2;16:988015. doi: 10.3389/fnana.2022.988015 (PMC9479637; doi:10.3389/fnana.2022.988015)
Supplement: Supplementary file 1 [file Data_Sheet_1.docx]

# Supplementary material

**Supplementary Figure 1. Cyclical pattern of causal interaction between PMCo and AOB for the rest of the studied stimuli.** (A) Analysis of the causal interaction for clean bedding. Right, Boxplot of causality peaks in both directions on consecutive epochs for diverse exploration segments. Note the different positions of the aligned orange and green boxplot as indicative of the difference between causal peaks for both directions. Left, Boxplot of the differences between causal peaks derived from right. Boxes represent the difference between consecutive peaks of causality. The orange line is intended to illustrate the cyclic progress of the difference in the causal relationship between PMCo and AOB. The comparisons between peaks evidence significant differences (peaks 1-2, p = 0.0275; peaks 3-4, p = 0.0061) (B) Analysis of the causal interaction for geraniol-scented bedding (peaks 1-2, p = 0.2810). Right and left show the same plots as in A. (C) Analysis of the causal interaction for castrated male-soiled bedding (peaks 1-2, p = 0.3141; peaks 3-4, p = 0.1910). Right and left show the same plots as in A. (D) Analysis of the causal interaction for female-soiled bedding (peaks 1-2, p = 0.2945; peaks 3-4, p = 0.3706). Right and left show the same plots as in A.

**Supplementary table 1.** Comparison of the proportion of theta cycles coupled to nested components before and after neutral or conspecific stimuli presentation (first to fourth minute) for each nucleus, corresponding to Figure 4. Wilcoxon test (W) and associated p-value (p), with significant p-values in bold.

| **Nucleus** | **Stimulus** | **1^st^ min** | | **2^nd^ min** | | **3^rd^ min** | | **4^th^ min** | | |
| --- | --- | --- | --- | --- | --- | --- | --- | --- | --- | --- |
|  |  | **W** | **p** | **W** | **p** | **W** | **p** | **W** | **p** |  |
| AOB | Neutral | 42.5 | 0.551 | 28 | 0.132 | 13 | **0.025** | 35 | 0.285 |  |
|  | Conspecific | 102 | 0.658 | 108 | 0.821 | 74.5 | 0.263 | 56.5 | 0.073 |  |
| MOB | Neutral | 40 | **0.048** | 46.5 | 0.094 | 44 | 0.074 | 36.5 | 0.062 |  |
|  | Conspecific | 39.5 | **3·10^-4^** | 45 | **5·10^-4^** | 39 | **5·10^-4^** | 68 | **0.004** |  |
| Me | Neutral | 25 | 0.091 | 16.5 | **0.026** | 11 | **0.018** | 5.5 | **0.004** |  |
|  | Conspecific | 28 | **0.002** | 36 | **0.006** | 21 | **0.002** | 25 | **0.001** |  |
| PMCo | Neutral | 12 | **0.009** | 10 | **0.005** | 18 | **0.029** | 20.5 | **0.048** |  |
|  | Conspecific | 2 | **8·10^-5^** | 0 | **9·10^-7^** | 11 | **2·10^-4^** | 14 | **4·10^-4^** |  |

**Supplementary table 2.** Comparison of the proportion of theta cycles coupled to nested components before and after neutral stimuli presentation (first to fourth minute) for each nucleus and each spectral component, corresponding to Figure 5. Student t-test (t) and associated p-value (p), with significant p-values in bold.

| **Nucleus** | **tSC** | **1^st^ min** | | **2^nd^ min** | | **3^rd^ min** | | **4^th^ min** | |
| --- | --- | --- | --- | --- | --- | --- | --- | --- | --- |
|  |  | **t** | **p** | **t** | **p** | **t** | **p** | **t** | **p** |
| **AOB** | 1 | -2.744 | **0.014** | -3.095 | **0.007** | -2.076 | 0.053 | -1.768 | 0.095 |
|  | 2 | -1.691 | 0.109 | -2.000 | 0.062 | -1.996 | 0.062 | -1.013 | 0.325 |
|  | 3 | -1.364 | 0.190 | -1.657 | 0.116 | -1.911 | 0.073 | -0.996 | 0.333 |
|  | 4 | 0.268 | 0.792 | -0.797 | 0.436 | -0.575 | 0.573 | -0.958 | 0.352 |
|  | 5 | 1.681 | 0.111 | 0.106 | 0.917 | -0.491 | 0.630 | 0.429 | 0.673 |
| **MOB** | 1 | -3.644 | **0.002** | -2.744 | **0.014** | -2.022 | 0.059 | -3.603 | **0.002** |
|  | 2 | -0.538 | 0.598 | 0.234 | 0.818 | 0.237 | 0.815 | -0.083 | 0.935 |
|  | 3 | 0.309 | 0.761 | -0.441 | 0.665 | -1.859 | 0.081 | -1.016 | 0.324 |
|  | 4 | 1.369 | 0.189 | -0.394 | 0.698 | -1.526 | 0.145 | -1.416 | 0.175 |
|  | 5 | 2.171 | **0.044** | -0.718 | 0.483 | 0.019 | 0.985 | -0.024 | 0.841 |
| **Me** | 1 | -2.048 | 0.056 | -2.886 | **0.010** | -1.549 | 0.140 | -2.767 | **0.013** |
|  | 2 | -1.935 | 0.070 | -3.041 | **0.007** | -1.646 | 0.118 | -2.735 | **0.014** |
|  | 3 | 0.053 | 0.959 | -2.074 | 0.054 | -1.794 | 0.091 | -0.824 | 0.421 |
|  | 4 | 1.629 | 0.122 | 0.785 | 0.443 | -0.820 | 0.423 | -1.078 | 0.296 |
|  | 5 | 1.548 | 0.140 | -0.389 | 0.702 | -0.959 | 0.351 | -2.323 | **0.033** |
| **PMCo** | 1 | -2.768 | **0.013** | -2.684 | **0.016** | -2.068 | 0.054 | -2.225 | **0.040** |
|  | 2 | -1.976 | 0.065 | -2.435 | **0.026** | -2.515 | **0.022** | -2.375 | **0.030** |
|  | 3 | -2.703 | **0.015** | -2.468 | **0.024** | -1.851 | 0.082 | -2.206 | **0.041** |
|  | 4 | -1.356 | 0.193 | -2.242 | **0.039** | -1.632 | 0.121 | -2.184 | **0.043** |
|  | 5 | -0.082 | 0.423 | -1.290 | 0.214 | -1.663 | 0.115 | -1.244 | 0.230 |

**Supplementary table 3.** Comparison of the proportion of theta cycles coupled to nested components before and after neutral stimuli presentation (first to fourth minute) for each nucleus and each spectral component, corresponding to Figure 6. Student t-test (t) and associated p-value (p), with significant p-values in bold.

|  | **Nucleus** | **tSC** | **1^st^ min** | | **2^nd^ min** | | **3^rd^ min** | | **4^th^ min** | |
| --- | --- | --- | --- | --- | --- | --- | --- | --- | --- | --- |
|  |  |  | **t** | **p** | **t** | **p** | **t** | **p** | **t** | **p** |
|  | **AOB** | 1 | -3.152 | **0.004** | -4.113 | **3·10^-4^** | -3.175 | **3·10^-3^** | -4.785 | **5·10^-5^** |
|  |  | 2 | -2.231 | **0.034** | -1.036 | 0.310 | -0.944 | 0.354 | -1.789 | 0.085 |
|  |  | 3 | 0.282 | 0.780 | 0.544 | 0.591 | -0.307 | 0.761 | -0.040 | 0.968 |
|  |  | 4 | 1.453 | 0.158 | 0.754 | 0.457 | 0.750 | 0.460 | 0.062 | 0.951 |
|  |  | 5 | 0.149 | 0.883 | -1.127 | 0.270 | -1.645 | 0.112 | -3.345 | **0.003** |
|  | **MOB** | 1 | -4.282 | **2·10^-4^** | -6.507 | **6·10^-7^** | -5.416 | **1·10^-5^** | -5.868 | **3·10^-6^** |
|  |  | 2 | -2.246 | **0.033** | -2.305 | **0.029** | -1.274 | 0.214 | -0.689 | 0.497 |
|  |  | 3 | -1.639 | 0.113 | -1.534 | 0.137 | -1.988 | 0.057 | -2.902 | **0.007** |
|  |  | 4 | 0.276 | 0.784 | -0.802 | 0.430 | -1.705 | 0.100 | -1.932 | 0.064 |
|  |  | 5 | -0.519 | 0.608 | -2.286 | **0.031** | -1.372 | 0.182 | -2.083 | **0.047** |
|  | **Me** | 1 | -3.849 | **0.001** | -4.230 | **2·10^-4^** | -2.825 | **0.009** | -3.542 | **0.002** |
|  |  | 2 | -2.762 | **0.010** | -2.651 | **0.013** | -2.782 | **0.010** | -3.097 | **0.005** |
|  |  | 3 | -2.257 | **0.033** | -2.984 | **0.006** | -2.404 | **0.024** | -3.117 | **0.004** |
|  |  | 4 | 0.084 | 0.934 | -1.132 | 0.268 | -2.070 | **0.048** | -2.862 | **0.008** |
|  |  | 5 | -0.947 | 0.352 | -2.504 | **0.019** | -2.134 | **0.042** | -2.917 | **0.007** |
|  | **PMCo** | 1 | -2.752 | **0.011** | -4.355 | **1·10^-4^** | -2.081 | **0.047** | -1.978 | 0.059 |
|  |  | 2 | -3.277 | **0.003** | -3.390 | **0.002** | -2.262 | **0.032** | -1.886 | 0.071 |
|  |  | 3 | -1.900 | 0.069 | -1.797 | 0.084 | -1.034 | 0.310 | -1.603 | 0.121 |
|  |  | 4 | -3.254 | **0.003** | -2.705 | **0.012** | -2.263 | **0.032** | -2.121 | **0.044** |
|  |  | 5 | -3.569 | **0.001** | -2.940 | **0.007** | -3.426 | **0.002** | -3.789 | **0.001** |

**Supplementary table 4.** Pearson’s correlations among the whole variable set, comparing altogether c-Fos expression in all nuclei and exploratory behavior. For each comparison Pearson's correlation coefficient (r) and p-value (p) are indicated. Significant correlations are highlighted in bold and shaded in light blue (p < 0.05). To facilitate the interpretation of the table, systems are colored following the same color code as the figures (vomeronasal in yellow, olfactory in magenta and reward system in green). **Abbreviations**: see Figure 9.

| **AOB** | **MeA** | **MePD** | **MePV** | **BAOT** | **PMCo** | **AAV** | **BSTmpm** | **MOB** | **Pir** | **CxA** | **ACo** | **NLOT** | **cAcb** | **sAcb** | **VTA** | **BLA** | **BMA** | **CPu** |  |  |
| --- | --- | --- | --- | --- | --- | --- | --- | --- | --- | --- | --- | --- | --- | --- | --- | --- | --- | --- | --- | --- |
| 0.402 | 0.174 | **0.655** | 0.542 | 0.353 | 0.589 | **0.699** | **0.641** | 0.147 | 0.084 | 0.584 | -0.025 | -0.158 | 0.322 | 0.057 | 0.243 | -0.037 | 0.210 | -0.094 | *r* | **Behavior** |
| 0.250 | 0.630 | **0.040** | 0.105 | 0.316 | 0.073 | **0.025** | **0.046** | 0.685 | 0.818 | 0.076 | 0.946 | 0.663 | 0.365 | 0.876 | 0.499 | 0.919 | 0.561 | 0.797 | *p* |  |
|  | 0.353 | 0.396 | 0.539 | 0.487 | 0.622 | 0.303 | 0.424 | 0.350 | 0.262 | **0.637** | 0.605 | 0.280 | 0.200 | 0.201 | -0.396 | -0.560 | -0.109 | 0.027 | *r* | **AOB** |
|  | 0.317 | 0.258 | 0.108 | 0.154 | 0.055 | 0.394 | 0.222 | 0.321 | 0.465 | **0.047** | 0.064 | 0.433 | 0.580 | 0.578 | 0.258 | 0.092 | 0.765 | 0.940 | *p* |  |
|  |  | **0.660** | **0.866** | **0.688** | **0.705** | 0.550 | 0.290 | **0.857** | 0.235 | **0.771** | 0.316 | 0.452 | 0.302 | 0.274 | 0.152 | 0.068 | 0.539 | -0.409 | *r* | **MeA** |
|  |  | **0.038** | **0.001** | **0.028** | **0.023** | 0.099 | 0.417 | **0.002** | 0.514 | **0.009** | 0.374 | 0.190 | 0.396 | 0.444 | 0.676 | 0.853 | 0.108 | 0.240 | *p* |  |
|  |  |  | **0.880** | 0.632 | **0.852** | **0.930** | 0.294 | 0.624 | 0.439 | **0.754** | 0.341 | 0.204 | 0.567 | 0.540 | 0.396 | 0.120 | **0.773** | -0.062 | *r* | **MePD** |
|  |  |  | **0.001** | 0.050 | **0.002** | **9·10^-5^** | 0.410 | 0.054 | 0.204 | **0.012** | 0.335 | 0.572 | 0.087 | 0.107 | 0.258 | 0.741 | **0.009** | 0.865 | *p* |  |
|  |  |  |  | **0.640** | **0.896** | **0.757** | 0.439 | **0.846** | 0.289 | **0.864** | 0.444 | 0.398 | 0.513 | 0.389 | 0.291 | 0.040 | 0.616 | -0.209 | *r* | **MePV** |
|  |  |  |  | **0.046** | **4.5·10^-4^** | **0.011** | 0.204 | **0.002** | 0.418 | **0.001** | 0.199 | 0.255 | 0.129 | 0.267 | 0.415 | 0.913 | 0.058 | 0.562 | *p* |  |
|  |  |  |  |  | **0.697** | 0.476 | 0.159 | 0.410 | 0.230 | **0.793** | 0.364 | 0.425 | 0.379 | 0.309 | -0.032 | -0.235 | 0.449 | -0.022 | *r* | **BAOT** |
|  |  |  |  |  | **0.025** | 0.164 | 0.661 | 0.239 | 0.523 | **0.006** | 0.301 | 0.221 | 0.280 | 0.385 | 0.929 | 0.513 | 0.193 | 0.953 | *p* |  |
|  |  |  |  |  |  | **0.730** | 0.445 | 0.599 | 0.346 | **0.925** | 0.539 | 0.457 | **0.651** | 0.407 | 0.035 | 0.086 | 0.573 | 0.049 | *r* | **PMCo** |
|  |  |  |  |  |  | **0.016** | 0.198 | 0.067 | 0.327 | **1.2·10^-4^** | 0.108 | 0.184 | **0.042** | 0.243 | 0.924 | 0.814 | 0.084 | 0.892 | *p* |  |
|  |  |  |  |  |  |  | 0.454 | 0.490 | 0.409 | **0.682** | 0.163 | 0.045 | 0.470 | 0.520 | 0.364 | 0.233 | 0.619 | -0.208 | *r* | **AAV** |
|  |  |  |  |  |  |  | 0.188 | 0.151 | 0.241 | **0.030** | 0.652 | 0.903 | 0.171 | 0.124 | 0.302 | 0.518 | 0.057 | 0.564 | *p* |  |
|  |  |  |  |  |  |  |  | 0.181 | -0.067 | 0.606 | 0.029 | 0.127 | 0.210 | 0.058 | 0.019 | 0.033 | -0.192 | -0.230 | *r* | **BSTmpm** |
|  |  |  |  |  |  |  |  | 0.617 | 0.853 | 0.063 | 0.936 | 0.727 | 0.560 | 0.874 | 0.959 | 0.929 | 0.595 | 0.522 | *p* |  |
|  |  |  |  |  |  |  |  |  | 0.025 | 0.603 | 0.489 | 0.480 | 0.400 | 0.359 | 0.334 | 0.132 | 0.484 | -0.405 | *r* | **MOB** |
|  |  |  |  |  |  |  |  |  | 0.945 | 0.065 | 0.152 | 0.160 | 0.252 | 0.308 | 0.346 | 0.717 | 0.157 | 0.246 | *p* |  |
|  |  |  |  |  |  |  |  |  |  | 0.141 | -0.039 | -0.270 | -0.168 | 0.197 | -0.087 | -0.308 | 0.484 | 0.199 | *r* | **Pir** |
|  |  |  |  |  |  |  |  |  |  | 0.698 | 0.916 | 0.450 | 0.642 | 0.585 | 0.812 | 0.386 | 0.156 | 0.582 | *p* |  |
|  |  |  |  |  |  |  |  |  |  |  | 0.497 | 0.536 | 0.602 | 0.375 | 0.001 | 0.037 | 0.380 | -0.118 | *r* | **CxA** |
|  |  |  |  |  |  |  |  |  |  |  | 0.144 | 0.110 | 0.066 | 0.286 | 0.999 | 0.918 | 0.279 | 0.745 | *p* |  |
|  |  |  |  |  |  |  |  |  |  |  |  | **0.823** | **0.720** | **0.679** | -0.053 | -0.038 | 0.179 | 0.376 | *r* | **ACo** |
|  |  |  |  |  |  |  |  |  |  |  |  | **0.003** | **0.019** | **0.031** | 0.884 | 0.916 | 0.621 | 0.285 | *p* |  |
|  |  |  |  |  |  |  |  |  |  |  |  |  | **0.755** | 0.597 | 0.078 | 0.210 | 0.202 | 0.304 | *r* | **NLOT** |
|  |  |  |  |  |  |  |  |  |  |  |  |  | **0.012** | 0.068 | 0.831 | 0.561 | 0.577 | 0.393 | *p* |  |
|  |  |  |  |  |  |  |  |  |  |  |  |  |  | **0.718** | 0.337 | 0.476 | 0.483 | 0.383 | *r* | **cAcb** |
|  |  |  |  |  |  |  |  |  |  |  |  |  |  | **0.019** | 0.341 | 0.164 | 0.157 | 0.274 | *p* |  |
|  |  |  |  |  |  |  |  |  |  |  |  |  |  |  | 0.410 | 0.190 | 0.481 | 0.375 | *r* | **sAcb** |
|  |  |  |  |  |  |  |  |  |  |  |  |  |  |  | 0.239 | 0.599 | 0.160 | 0.285 | *p* |  |
|  |  |  |  |  |  |  |  |  |  |  |  |  |  |  |  | 0.287 | 0.557 | 0.071 | *r* | **VTA** |
|  |  |  |  |  |  |  |  |  |  |  |  |  |  |  |  | 0.422 | 0.095 | 0.846 | *p* |  |
|  |  |  |  |  |  |  |  |  |  |  |  |  |  |  |  |  | 0.310 | -0.118 | *r* | **BLA** |
|  |  |  |  |  |  |  |  |  |  |  |  |  |  |  |  |  | 0.383 | 0.745 | *p* |  |
|  |  |  |  |  |  |  |  |  |  |  |  |  |  |  |  |  |  | 0.167 | *r* | **BMA** |
|  |  |  |  |  |  |  |  |  |  |  |  |  |  |  |  |  |  | 0.645 | *p* |  |


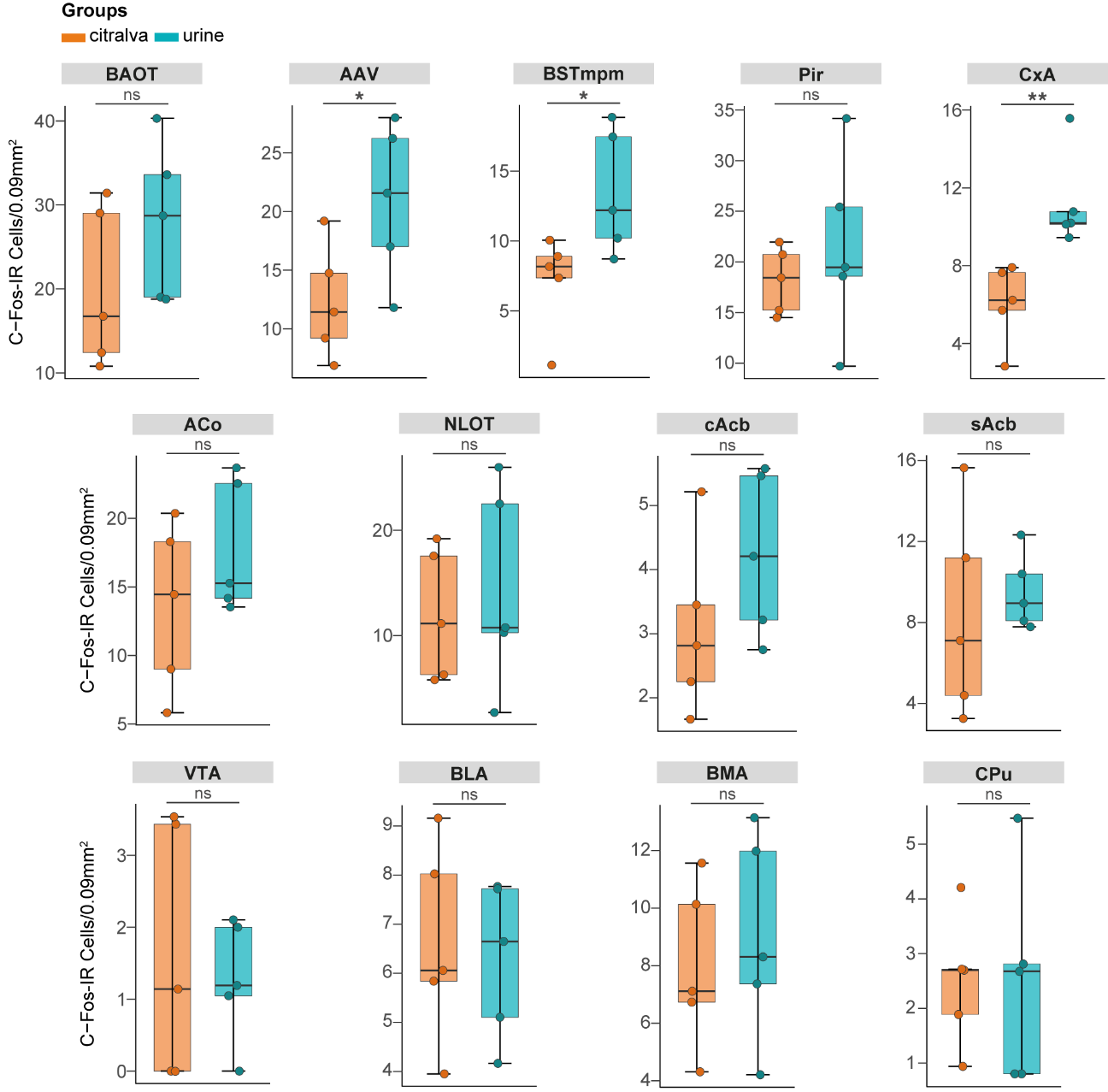


**Supplementary Figure 2. Box plots graphically representing c-Fos cell density data distribution in the nuclei not shown in Figure 9B.** Group data distribution comparing cell activation density (c-Fos-immunoreactive cells/0.09 mm^2^) between citralva (orange, n = 5) and urine (blue, n = 5) groups. Box plots are defined in terms of minima and maxima by whiskers, and the center and bounds of box by quartiles (Q1–Q3). Two-sided t or Mann-Whitney U test: *p < 0.05; **p < 0.01. **Abbreviations**: **BAOT**, bed nucleus of the accessory olfactory tract; **AAV**, anteroventral anterior amygdaloid nucleus; **BSTmpm**, medial posteromedial bed nucleus of the stria terminalis; **Pir**, piriform cortex; **CxA**, cortex amygdala transition area; **ACo**, anterior cortical amygdaloid nucleus; **NLOT**, nucleus of the lateral olfactory tract; **cAcb**, nucleus accumbens core; **sAcb**, nucleus accumbens shell; **VTA**, ventral tegmental area; **BLA**, basolateral amygdaloid nucleus; **BMA**, basomedial amygdaloid nucleus; **CPu**, caudate putamen (striatum).
